# Supplementary material for: Comparative analysis of three studies measuring fluorescence from engineered bacterial genetic constructs
Source: PLoS One. 2021 Jun 7;16(6):e0252263. doi: 10.1371/journal.pone.0252263 (PMC8183995; doi:10.1371/journal.pone.0252263)
Supplement: S1 File — List of 2708 additional consortium authors comprising the iGEM Interlab Study Contributors. (PDF) [file pone.0252263.s001.pdf]

S1 File: Consortium Author List

Jacob Beal, Geoff S. Baldwin, Natalie G. Farny, Markus Gershater,
Traci Haddock-Angelli, Russell Buckley-Taylor, Ari Dwijayanti, Daisuke Kiga,
Meagan Lizarazo, John Marken, Kim de Mora, Randy Rettberg,
Vishal Sanchania, Vinoo Selvarajah, Abigail Sison, Marko Storch,
Christopher T. Workman, and the iGEM Interlab Study Contributors

Consortium authors include all persons identified by contributing teams as deserving co-authorship credit. Contributors are listed alphabetically within team, and teams alphabetically. Team names are given as identified in iGEM records: full details of each team's institution and additional members may be found online in the iGEM Foundation archives at:

`http://`*year*`.igem.org/Team:`*name*

e.g.: full information on the 2016 ETH_Zurich team may be found at:

`http://2016.igem.org/Team:ETH_Zurich`

In total, there are 2708 consortium authors in the iGEM Interlab Study Contributors.

2016 Teams

The iGEM 2016 Interlab Contributors comprise a total of 411 authors from 72 institutions.

- **Aachen:** Volkan Besirlioglu, Carolina Bonerath, Vroni Czotscher, Alexander Deitert, Annika Graeve, Andrea Hoeltken, Carsten Ludwig, Svenja Meyer, Sujeethkumar Prithiviraj, Viviane Schink, Katja Schroder, Prannoy Seth, Niharika Singhal, Lea Steinbeck, Martin Thiele, Till Tiso, Praveen Iyyappan Valsala, Zibo Wei
- **Aalto-Helsinki:** Emilia Broman, Hele Haapaniemi, Saara Hiltunen, Rashad Ismayilzada, Laura Laiho
- **Aix-Marseille Universite:** Gael Chambonnier, Louis Givelet, Sandra Michel-Souzy, Tangui Messina, Claire Raynaud, James Sturgis
- **Alverno_CA:** Monica Barsever, Samuel Clamons, Victoria Chen, Asia Leong, Melody Wu
- **Austin_UTexas:** Daniel Garza, Dennis Michael Mishler
- **BIT:** Daoyin Chen, Zitong Wang, Wei Yang, Jinfu Zhang, Chuangui Zhou
- **BostonU:** Jeffrey M. Marano, Benjamin H. Weinberg
- **CGU_Taiwan:** Yu-chang Ku, Bin-Tse Lin, Yaa-Jyuhn Meir, Yu Yan Jessie Wang
- **CSU_Fort_Collins:** Madeline Cox, Sharon Lian, Casey Mazzotta, Dylan Miller, Christie Peebles, Courtney Robertson, Alec Schmutte, Allison Zimont
- **DTU-Denmark:** Line Andresen, Trine Sofie Bladt, Tobias Bertram Petersen, Stefani Pjaca, Christopher T. Workman
- **Edinburgh_OG:** Heather Baker, Elvis Bernard, Chris French, Jon Marles-Wright, Yaiza Velasquez
- **Edinburgh_UG:** Heather Barker, Aitor De Las Heras, Azzurra De Pace, Peter Iliev, Rosie Maddock

- **ETH_Zurich:** Raffaele Altamura, Asli Azizoglu, Yaakov Benenson, Daniel Gerngross, Mattia Gollub, Verena Jaeggin, Janina Linnik, Telma Lopes, Claude Lormeau, Sven Panke, Lucas Schaus, Lukas Schmidheini, Sophie Skriabine, Joerg Stelling, Tina Subic, Andreas Zingg
- **Evry:** Vincent Gureghian, Cecile Jacry
- **Exeter:** Dan Barber, Joel Burton-Lowe, Jack Fleet, Jamie Gilman, Eloise Lloyd, Hannah Osborne, Emily Reeves, Pablo Sharrock
- **Georgia_State:** Amy Andrelchik, Matthew Brewer, Cara A Jones, Nia Kapitanova, John Richardson, Debby Walthall, Joseph Whitley
- **Gifu:** Takema Hasegawa, Tatsuki Isogai, Tomoya Kozakai, Haruka Maruyama, Akihiro Moriyama, Ryo Niwa, Seita Tomida
- **Glasgow:** James Provan, Matej Vucak
- **Hannover:** Severin Hachenberg, Ann-Katrin Hannemann, Wiebke Juenemann, Sabine Klatt, Kim Luehmann, Bianca Naumann, Louise Schaar, Janis O. Schleusner, Martin Singer, Theresa Schmidt, Arthur Ulmann, Roman Zimbelmann
- **Hong_Kong_HKUST:** Kar On Cheng
- **HUST-China:** Zhangyu Cheng, Lin Di, Wangjie Liu
- **HZAU-China:** Pan Chu, Wenqi Huang, Zhujun Xia, Boyao Zhang, Anqi Zhou
- **IISc_Bangalore:** Aneesh Baburaj, Arunavo Chakraborty, Ayan Das, Prabaha Gangopadhyay, Shreyas Gopalakrishnan, Abhijeet Krishna, Aiswarya Prasad, Deepak Kumar Saini, Umesh Varshney
- **IIT_Kharagpur:** Chetan Khandelwal, Pruthvi Patel, Rhushikesh Phadke, Sanjay Prasad
- **IIT-Madras:** Nikunj Mehta, Shashi Bala Prasad, Nitish Kumar Singh, Venkata Subrahmanyan
- **INSA-Lyon:** Delphine Bourgeon, Margaux Poulalier-Delavelle, Paul Zanoni
- **Jilin_China:** Luwei Huang, Yuwei Huang, Tingtao Wu, Mingjun Zhang
- **Leiden:** Lizah van der Aart, Vincent de Bakker, Valentijn Broeken, Lucie Delfos, David van Driel, Koen Hokke, Wouter Liefting, Lisanne van Oosterhoud, Frans Rodenburg, Sjoerd Seekles, Max Snijders, Charlotte van de Velde, Lisa Verbeij, Guus de Wit
- **LMU-TUM_Munich:** Stefan Achatz, Volker Morath, Arne Skerra, Manuel Trauner
- **Macquarie_Australia:** Louise Jennifer Brown, Yeo Jin Cheong, Elizabeth Daniel, Michael Gibbs, Erwin Da Silva Gruener, Thi Huynh, Edward Moh, Rizell Ronan, Aleen Juma, Jayden Rouse, Robert Willows
- **MIT:** Nicholas DeLateur, Colleen Foley, Brian Teague
- **Newcastle:** Jake Burton, Olliver Burton, Emilija Kopustaite, Kerry Lewis, Kristina Marko, Lauren Mills, Josh Rushby, Rupert Truman
- **NKU_China:** Liangti Dai, Xiao Liu, Xinhao Song, Yuxiu Xiong
- **Northwestern:** Michelle Cai, Sam Davidson, Jordan Harrison, John Mordacq, Paul Perkovich, Kelly Schwarz
- **NTU-Singapore:** Zhehui Liu, Kean Hean Ooi, Meng How Tan, Jing Yen Yong

- **NYU-AD:** May Baho, Christine Chung, Veronika Li, Alejandra Trejo
- **Oxford:** Julia Davis, Iain Dunn, Andreas Hadjicharalambous, Chris Jones, George Wadhams
- **Paris_Bettencourt:** Mislav Acman, Jason Bland, Nadine Bongaerts, Alicia Pereira e Calvo-Villamanan, Sebastian Sosa Carrillo, Sebastien Gaultier, Allison Bricknell George, Ibrahim Haouchine, Elisa Hubert, Mani Sai Suryateja Jammalamadaka, Ariel Lindner, Thomas Meiller-Legrand, Shruthi Narayanan, Antoine Poirot-Bourdain, Antoine Villa, Edwin Wintermute
- **Paris_Saclay:** Mahnaz Sabeti Azad, Caroline Correia, Lea Talbot, Charlene Valadon
- **Peking:** Li Cheng, Dong Yiming
- **Pittsburgh:** Claire C. Chu
- **Purdue:** Aditi Acharya, Sohinee Bera, Alyson Chaney, Emma Foster, Jenna L. Rickus, Kevin Solomon
- **SDSZ_China:** Xinyi Ma, Hetian Su
- **ShanghaitechChina:** Fang Ba, Yi Liu, Qiaoqi Wang, Chao Zhong, Haolong Zhu
- **Stanford-Brown:** Michael Becich, Charles Gleason, Julia Gross, Cynthia Hale-Phillips, Anna Le, Eric Liu, Taylor Pullinger, Elias Robinson, Theresa Sievert, Taylor Sihavong, Gordon Sun, Amy Weissenbach
- **Stony_Brook:** Jarrod French, Ryan Kawalerski, Daniel Thach
- **SUSTech_Shenzhen:** Shaohua Ling, Shixin Lu, Qiao Ye, Fei Zeng
- **SVCE_CHENNAI:** Samudra, Varshni, Priyadarshni, Yashashwini, Veveeyan, Vidya, Mamta, Karthik, Ramit, Hariharan, Nalinkanth V Ghone, Arun Kumar, Jayaprakash
- **Sydney_Australia:** Nicholas Coleman, Claudia Moratti
- **SYSU-CHINA:** Diao Liu, Xiaowen Mao, Chunyang Ni, Yu Tao
- **SYSU-Software:** Minxue Liu, Ren Liu, Baiyu Wang, Xi Wang, Le Yu, Dongyu Zeng, Yujie Zhong
- **TEC-Costa_Rica:** Samantha Garcia, Rafael Montenegro, Paula Thiel, Pablo Vargas, Osvaldo Vega, Sofia Vieto
- **TU_Delft:** Anne Meyer, Dominik Schmieden, Tessa Vergroesen, Maria Vazquez Vitali
- **TU-Eindhoven:** Luc Brunsveld, Tom de Greef, Stijn Hofstraat, Vera Koomen, Carolien de Korte, Marijn Kruit, Nynke de Leeuw, Maarten Merkx, Laura Rijns, Tom van Sonsbeek, Bram van der Velden, Rene Verhoef
- **Tuebingen:** Lukas Fuhs, Nikolas Layer, Yana Parfyonova, Katharina Sporbeck
- **UCC_Ireland:** Brandon Malone, Kevin Ryan, Amy Bergin, Seema Subedi, Regina Walsh
- **UCLA:** Megan A. Satyadi
- **UESTC-China:** Ruining Cai, Chongwen Liang, Huan-Huan Wei
- **UIUC_Illinois:** Caroline Blassick, Viraat Goel, Auinash Kalsotra, Augustine Koh, Ting Lu, Mariam Saadah
- **UMaryland:** Narendranath Bhokisham, Seth Cohen, Paula Kleyman, Chun Mun Loke, Jacob Premo, Chaoyang Wang, Nancy Zhang

- **Uppsala:** Gunnar Johansson, Fredrik Lindeberg, Kristoffer Lundmark
- **USP-EEL-Brazil:** Leonardo de Oliveira Ferreira, Aline Larissa Gonçalves, Andre Tomas Vilela Hermann, Fernando Segato, Hemerson Sulpicio Junior
- **USP_UNIFESP-Brazil:** Victor Nunes de Jesus, João Vitor Dutra Molino, Cauã Antunes Westmann
- **Vanderbilt:** Ophir Ospovat, Jarrod Shilts
- **Vilnius-Lithuania:** Auguste Ambrazaite, Kotryna Cekuolyte, Eigile Eidenaite, Dovile Ezerskyte, Diana Ikasalaite, Jonas Juozapaitis, Laura Mataite
- **Warwick:** Liam Carroll, Lorna Flintham, Isobel Holden, Alfonso Jaramillo, Konstantina Koteva, Manish Kushwaha, Egheosa Ogbomo, Chinwe Odili, Anjana Radhakrishnan, Robert Richardson, William Rostain, Hayden Tobin
- **WashU_StLouis:** Drew Ells, Zach Glick, Harley Greene, Cheryl Immethun, Gillian Myers
- **William_and_Mary:** Kalen Clifton, Christine Gao, Andrew Halleran, Ethan Jones, Likhitha Kolla, Joseph Maniaci, John Marken, John Mitchell, Callan Monette, Adam Reiss, Margaret Saha, Gregory D. Smith
- **WPI_Worcester:** Natalie Farny, Frederick Gergits, Matthew Googins, Sarah Martin, Virginia Massa, Allison Van Fechtmann, Jingyi Wu
- **XJTLU-CHINA:** Mengjia Li, Tiancheng Li, Chengyang Nie, Lei Zhang, Qing Zhang
- **XMU-China:** Kainan Chen, Zeyue Gao

2017 Teams

The iGEM 2017 Interlab Contributors comprise a total of 898 authors from 188 institutions.

- **Aachen:** Volkan Besirlioglu, Stefanie Brands, Gerolamo Rota
- **Aalto-Helsinki:** Meo Ekroos, Maisa Vuorte
- **Aix-Marseille:** Lisa Frugoli
- **Albion_College:** Matt Anderson, Quenten Mahoney, Hernan Rico, Ken Saville, Emma Schlachter, Nicole Woodhead
- **Amazonas_Brazil:** Maria Clara Tavares Astolfi, Raquel Silva Bezerra, Marcelo Valente Pinto, Jerusa Araujo Quintao
- **Amsterdam:** Yuki Esser, Max Guillaume, Josine Oude Lohuis, Age Tjalma, Thijs van Schaik, Bram van de Putte
- **AQA_Unesp:** Mariana Biondi Cesar, Daniel Augusto Cozetto, Danielle Biscaro Pedrolli, Nathan Vinicius Ribeiro, Patrick Neves Squizato, Rafael Brull Tuma
- **AshesiGhana:** Miquilina Anagbah, Nana Oye Djan, Justice Essuman, Adam Ghaffar, Stephan Ofosuhene, Elena Rosca, David Sasu, Claude-Noel Tamakloe
- **ASTWS-China:** Sam Dong
- **Austin_UTexas:** Matthew Hooper, Ian Overman
- **Berlin_diagnostX:** Svenja Nierwetberg, Annkathrin Ruhm

- **BGIC-Union:** Liu Yu Han, Xiao Qi, Wang Bo Xiang, Xiao Zi Xin
- **Bielefeld-CeBiTec:** Maximilian Edich, Camilla Marz
- **Bilkent-UNAMBG:** Busra Nur At, Eray Ulas Bozkurt, Artun Bulbul, Ezgi Dikici
- **BNDS_China:** Hong Chen, Zhongxiu Hu, Zhuoyi Yang, Kuo Zhang, Yihao Zhang
- **BOKU-Vienna:** Michael Baumschabl, Mathias Gotsmy, Christina Hausjell, Felicia Hsu, Mina Modarressy, Lisa Papp, Rebecca Sagmeister
- **BostonU:** Thomas Costa, Saimrunali Dadigala, Alan Pacheco, Abigail Sasdelli, Madeline Simota, Stephen Tucker, Shuyi Xu
- **Botchan_Lab_Tokyo:** Kohei Homma, Rino Homma, Chiharu Ishikawa, Minami Nakayama, Ayumi Taga
- **Bristol:** Nick Comben, Sarah Cooke, Paul Curnow, Phoebe Dace, Sarah Deegan, Virginie Dufour, Ben Hardy, Jeremy Joannes, Jonathon De Oliveira, Albert Wigmore
- **British_Columbia:** Rodrigo Vallejos Chavez, Evan Gibbard, Christine Kim, Maria Mendizabal, Catherine Pan, Leif Pedersen
- **Cadets2Vets:** Ian Gutierrez, Kristina Ilyovska, Zoey Shisler, Brendan Studebaker
- **CCA_San_Diego:** Philippe Hansen-Estruch, Aida Razavilar
- **CCU_Taiwan:** Guo-En Chang, Fei Chen, Yi-Chen Hsieh, Cheng-Ping Jheng, Cheng-I Lee, Cheng-Yu Lee, Chung-Hao Lee, Zi-You Lin, Fang-Yi Lo, Yi-Chia Su, Thuan-Thien Tchen
- **CGU_Taiwan:** Chyun-Jye Lin, Yi-An Shih, Tsung-Lin Tsai, Hsiang-chia Yu
- **Chalmers-Gothenburg:** Sofie Gunnarsson, Amanda Ristinmaa, Tove Widen
- **CLSB-UK:** Adam Jones, Abe Tolley, Sasha Walter
- **CMUQ:** Mohamed Bouaouina, Dina Nayel, Saad Rasool, Annette Vincent
- **Cologne-Duesseldorf:** Marvin Hubert, Rene Inckemann
- **ColumbiaNYC:** Noah Basri, Brandon Cuevas, Jennifer Fang, Benjamin Greenfield, Alex Kim, Nathan Lian, Ross McBee, Panagiotis Oikonomou, Tarun Srinivasan
- **CPU_CHINA:** Liujun He, Xin Li, Sisi Qu, Tengjie Yu, Chu Zhang, Qihang Zhao, Zhili Zhao
- **CSMU_NCHU_Taiwan:** Wei-Hao Huang, Wei-Yang Lee, Chuan Li, Tin-Gyu Lin, Shao-Chi Lo, Rong-Tzong Tsai
- **CU_Boulder:** Maxwell Saal
- **Dalhousie:** Matthew Curry, Landon Getz
- **DEIAGRA:** Dipinte Gupta, Naziya Parveen, Mrinalini Prasad, Rajiv Ranjan, Richa, Gauri Sharma, Simran Singh, Pragati Yadav
- **DTU-Denmark:** Minh Chau Greulich, Philip Hau Sorensen, Andreas Treschow
- **ECUST:** Li Fengting, Liang Mindong, Chen Weijie, Jiang Zhenzhou
- **Edinburgh_OG:** Heather Barker, Ti He

- **Edinburgh_UG:** Pepe Cabezas, Teri Cheng, Ted Leung, Jack Suitor
- **Emory:** Mone Anzai, Davin Lama, Cyrillus Tan, Christian Vallejo, Daniel Won
- **EpiphanyNYC:** Yeji Cho
- **ETH_Zurich:** Vasileios Cheras, Lida Vadakumchery
- **Evry_Paris-Saclay:** Paul Del Rincon
- **Exeter:** Daniel Barber, Jamie Gilman, Karolina Kostrzynska, Louis Philipp Lukas, Rahan Nazeer, Kinga Zielinska
- **FAFU-CHINA:** Yi Cai, Haiyang Chen, Yanyun Zhang
- **Florida_Atlantic:** Erick Espana, Daniela Garzon-Aljure, Douglas Holmes, Mirjana Pavlovic, Rachel StClair, Arianna Staton
- **Freiburg:** Julius Holzschuh, Laura Neschen
- **FSU:** Megan Donnelly, Wes Kosater, Isabel Lamb, Damilola Ologunagba
- **Fudan:** Ao Feng
- **Fudan_China:** Hanzhen Zhang
- **Gifu:** Tatsuki Isogai
- **Glasgow:** Sean Colloms, James Provan, Hannah Taylor
- **Greece:** Arampatzis Asterios, Giannitsis Charilaos, Sandaltzopoulou Elissavet, Ballhysa Eugen, Nikolopoulos Thomas
- **Groningen:** Mart W. Bartelds, Thijs W. Ettema, Matthijs de Koning
- **GZHS-United:** Qiyuan Chen, Yinghui Wang
- **Hamburg:** Andreas Czech, Ruth Rietow Fluorescein, Guillermo Molina, Alina Nicolai, Elisabeth Orlowski, Shanti Ricke, Annika Soltau
- **Harvard:** Hyeon-Jae Seo
- **HBUT-China:** Ning Dai, Zhuyu Han, Wenwen Liu
- **Heidelberg:** Lukas Adam, Julius Upmeier zu Belzen, Thore Buergel, Catharina Gandor, Marita Juliane Klein, Jan Mathony, Pauline Luise Pfuderer, Lukas Platz, Moritz Przybilla, Max Schwendemann
- **HFLS_H2Z_Hangzhou:** Jiayue Guo, Jianan Li, Qingrui Sun, Shuyun Zhang
- **Hong_Kong_HKU:** Ravneet Saran, Yash Shukla, Tsang To
- **Hong_Kong_HKUST:** Tsz Man Cheung, Yuen Tung Li, Belkis Chau Zhong
- **Hong_Kong-CUHK:** Chun Sing Chow, Ching Yuet To
- **HUST-China:** Long Cheng, Haibo Huang, Shaofeng Liao, Huiping Shi, Efan Wang, Ziyang Xiao, Kangyuan Yu
- **HZAU-China:** Songtao Cheng, Zhiqing Guo, Yini Miao, Qiqin Mo, Zhujun Xia, He Yang, Yinqing Zen, Lingyu Zhong, Anqi Zhou

- **ICT-Mumbai:** Shalini Deb, Aditya Kamat, Arvind M. Lali, Revathi Reddy, Shamlan M. S. Reshamwala
- **IISc-Bangalore:** Kunal Helambe
- **IISER-Mohali-INDIA:** Prateek Chawla, Ashwin Kumar, Bhupinder Singh, Piyush Yadav
- **IISER-Pune-India:** Chaitanya Athale, Kunalika Jain, Yash Jawale, Snehal Kadam, Aarti Kejriwal, Avani Koparkar, Zakhiya PC, Charvee Ravichandran, Tanmaya Sethi, Jyothish Sudhakaran
- **IIT_Delhi:** Divya Chaudhary, Siddhesh Gandhi, Sashi Kalan, Pratyush Maini, Abhilash Patel, Kshitij Rai, Saksham Sharma, Pulkit Srivastava
- **Jilin_China:** Yan Chen, Jeremy Gagne, Ming Han, Ali Hou, Cheng Hu, Zihan Lu, Shan Wang, Zihao Wang, Yang Zhan
- **JNFLS:** Na Su, Xinyu Zhou
- **Judd_UK:** Mateo Hoare, Nikita Shukan
- **KAIT_JAPAN:** Naho Koyama, Zhi Ling Lui
- **Kent:** Dan Brunkow, Laurens Heling, Neil Kad, Taylor Monaghan, Laulwa Al Salloum
- **Kobe:** Kyosuke Kita, Shoya Komura, Saki Matsui, Tomoya Nishiguchi, Momoko Okabe
- **KU_Leuven:** Imke Ensinck
- **Lambert_GA:** Gaurav Byagathvalli, Ellie Kim
- **Lanzhou:** He Jingdian, Zhang Shuting, Wu Taoping, Ren Yi, Jing Zhecheng, Lin Zhicheng, Huang Zonghui
- **Lethbridge:** Michelle Cradduck, Simmone D'souza, Regan Evanson, Cynthia Fonderson, Jonathan Hampshire, Taylor Sheahan, Dustin Smith, Sandhya Sunuwar, Kristi Turton, Hans-Joachim Wieden
- **LUBBOCK_TTU:** Angela Ahrens, Kenia Ascencio, Brittany Gaither, Brittney Hoang, Gregory Knox, Brandon Palomo
- **Lund:** Julia Dobrich, Malin Jonsson, Rebeka Kovacic, Frida Rosman, Adham Sakhnini, Peter Skog
- **Macquarie_Australia:** Ali Ali, Taylor Berry, India Boyton, Ari Edmonds, Simone Hargraves, Jocelyn Johns, Adrianna Minichiello, Winonah Riddell
- **Manchester:** Marc Biarnes-Carrera, Jessica Burns, James Engleback, Will Finnigan, Alice Fraser, Theodore Reinhard Glenaldo, Amber Hall, Adam Hannaford, Owen Jonathan Julianto, Ross Kent, Ong Jun Yang
- **ManhattanCol_Bronx:** Farzana Begum, Brian Evans, Bryan Wilkins
- **McMasterU:** Tony Chen, Angela Hong Tian Dong, Audrey Jong, Damian Tran
- **Mingdao:** Chi-Wen Chen, Li-Yun Chen, Yu-Chen Fang, Chen-His Shih, Yu-Hao Ting
- **Missouri_Rolla:** Ryan Baumann, Benjamin Bleitz, Kent Gorday
- **MIT:** Nia Myrie, Adil Yusuf
- **MSU-Michigan:** Brian Amburn, Noelia Barvo, Danny Ducat, Ciara Fromwiller, Bjoern Hamberger, Donna Liebelt, Cody Madsen, Michaela TerAvest, Serenity Tyll, Tim Whitehead

- **Munich:** Erika Chacin, Dawafuti Sherpa
- **NAU-CHINA:** Wei Wang, Jiang Yuqian
- **NAWI_Graz:** Andreas Berner, Martin Odabas, Henrik Seyfried, Agi Zoto
- **NEU-China:** Jiawen Yang, Ni Zong
- **Newcastle:** Michaela Chapman, Declan Kohl, Marcia Pryce, Shriansh Vyas, Anna Walsh, Evangeline Whittaker, Zoe Wilson
- **NJU-China:** Kaiming Li, Jincheng Wu, Yu Zhou
- **NKU_China:** Chenyue Guo, Xuncheng Su, Xin Wen, Yonghui Xie, Jiaxing Yang, Lingyang Zhang, Zhuochen Zhang
- **Northwestern:** Jack Arnold, Katerina Cheronis, Tyler Kramlich, Ayesha Rahman, Yunlu Sun
- **NPU-China:** Weifeng Lin, Yun Liu, Weiwei Qin, Zexun Wu, Bingzhao Zhuo
- **NTHU_Taiwan:** Chi-Hung Shu, Tina Yang
- **NTU_SINGAPORE:** Kasturi D/O Markandran, Shaw Kar Ming, Kelvin Suriyaputra, Danny Teo Shun Xiang
- **NU_Kazakhstan:** Saniya Aitmaganbetova, Lyazzat Bekish, Zhassulan Ispolov, Yersultan Mirasbekov, Assel Mukhanova, Sultan Mussakhan, Almira Zhantuyakova
- **NUDT_CHINA:** Xiaoju Chang, Shan Huang, Yilin Lei, Min Xiao, Qijie Xu, Junling Yang, Herui Zhang, Chushu Zhu
- **NUS_Singapore:** Maciej Holowko, Premkumar Jayaraman, Zhang Jingyun, Lee Hwee Siong Julian, Dong Thi Anh Khue, Chee Wai Kit, Poh Chueh Loo, Ren Meifeng, Lim Yan Ping, Wilbert Tan, Zheng Wenhao, Tian Zixu
- **NWU-CHINA:** Xu Han, Qinbo Zheng, Junjie Zhu
- **NYU_Shanghai:** Amy Lam, Honey Asrat Lera, Zhongling Liang, Ziyu Zhao
- **OUC-China:** Sunan Hu, Hao Jiang, Rong Mu, Lei Zhu
- **Oxford:** Nick Delalez, Jei Diwakar, Angela Hellyer, Arthur Norman
- **Pasteur_Paris:** Maxime Chazal, Pierre-Henri Commere, Diane Girard, Paul Jeammet, Nathan Ronceray, Sandrine Schmutz
- **Peking:** Chen Hong
- **Peshawar:** Shaheer Sabz Ali, Muhammad Dawood, Sarah Farooq, Muhammad Ismail, Moaz Khaliq, Hassnain Qasim
- **Pittsburgh:** Ian Carleton, Dorsin Chang, Gabby Gannon, Vivian Hu, Liam McFadden
- **Purdue:** Kevin Fitzgerald, Caleigh Roleck, Christopher Schorr
- **Queens_Canada:** Nolan Neville, Mady Thompson
- **REC-CHENNAI:** Amal Jude Ashwin F, Srisreya Madhala, Vasavi N S, Saranya V
- **RHIT:** Richard Anthony, Audrey Brand, Paul Earhart, Allison Harpel, Madison Muncie, Irene Reizman, Julia Walsh, Yifei Yang

- **Rice:** Catherine Dunaway, Anna Guseva, Jonathan Pan, David Yang, Matt Ykema
- **RPI_Troy_NY:** Krystyna Farrell, Douglas Meadow, Helen Merricks, Maily Nguyen, Blossom Wong
- **SCU_China:** Han Kang, Changhe Li, Tian Liu, Caiqin Wang, Fuqiang Yang, Yuanhan Yang, Chenming Zhang, Yanling Zhong
- **SCU-WestChina:** Jiusi Guo, Qingge Ma, Zheng Wang, Hongbin Yu
- **SCUT-China_A:** Rirong Hu, Mengjiao Ni, Baihua Wu
- **SCUT-FSE-CHINA:** Pan Qi, Liu Xiaojia, Huang Zisang
- **SDU_CHINA:** Ziming Chen, Zhuoxuan Jia
- **Shanghaitech:** Fang Ba, Mingzhe Chen, Xiangning Chen, Xue Fang, Bo Gao, Fang Luo, Yun Ni, Weiyi Tang, Yiqing Yang
- **SHSBNU_China:** Xiaoxiao Chen, Weihang Guo, Huanding Ji, Boxuan Li, Xuan Wang, Chen Zhang, Yihao Zhang, Haoyu Zhou
- **SSTi-SZGD:** Liangli Liu, Cheng Qiu, Xiaolin Wu
- **Stanford-Brown:** Katie Gu, Lynn J. Rothschild, Brian Vuong
- **Stockholm:** Aman Mebrahtu, Larsen Vornholz
- **Stony_Brook:** John Peter Gergen, Marvin O'Neal, Rideeta Raquib, Gene Yang
- **Stuttgart:** Heidi Muehl
- **SUIS_Alpha_Shanghai:** Jia Jun Gu, Zhengzhou Jiang, Jia Jin
- **SVCE_CHENNAI:** S C Azhagammal, Ramit B, Veveeyan S, Aditi Valecha
- **Sydney_Australia:** Emaleen Najjar, Courtney Pratt, Erica Stewart
- **SYSU-Software:** Yongjun Lu, Dong Shen, Yimin Zheng
- **TecCEM:** Itzel Becerril, M.C. Carlos Cruz, Luis Fernando Durán-Armenta, Ana Laura Torres Huerta, Jorge Ordóñez, Juan Carlos Rueda, Nicole Saucedo
- **TECHNION-ISRAEL:** Maya Engal, Nuphar Reinhardt, Alexey Tomsov
- **TokyoTech:** Hazuki Hasegawa, Kazuya Isawa, Hinako Kattoka, Kazunori Motai, Hikaru Nakaya, Tamon Sato, Moe Takahashi, Takuma Yasuo
- **Tongji_China:** Wei Zhu
- **Toronto:** Carla Hamady-Schroeder, Ahmed Ibrahim, Lindsey Kuramoto, Gurjit Mander, Tian Sang, Victor Xu, Amy Yeung
- **TP-CC_San_Diego:** Yunqing Gao
- **Tsinghua-A:** Zhaojian Wang
- **TU_Darmstadt:** Feodor Belov, Claudia Kreher, Thea Lotz, Tim Maier, Bea Marie Spiekermann, Beatrix Suss, Heribert Warzecha, Jennifer Zimmermann
- **TU_Dresden:** Vanessa Gilly, Philipp Popp, Lisa Schone

- **TU-Eindhoven:** Leon Haas, Sebastiaan Hamers, Ralf Philipsen, Marle Vleugels, Laura van Smeden, Nathalie van de Laar, Wytse van den Berg
- **TUDelft:** Kasper Spoelstra, Jochem Vink, Amaria Vledder
- **Tuebingen:** Marcel Conrady, Milena Krach, Michael Krummhaar, Brian Weidensee, Fan Zhang
- **UAlberta:** Ethan Agena, Heather Baker, Robert E. Campbell, Rochelin Dalangin, Farynna Facundo, David Herczeg, Teresa Nguyen-Pham, Monica Takla
- **UC_San_Diego:** Yansen Geng, Jieting Hu
- **UCalgary:** Amy Chen, Jacob Grainger, Deirdre Lobb, Sam Wilton-Clark
- **UCAS:** Li Yunfan
- **UCC_Ireland:** Ellen Byrne, Chloe Darragh-Hickey, Eoin Hurley, Daniel Moore, Sumitha Grace Pandiaraja
- **UCL:** Rosalia Cardos, Nuria Codina, Alexander Cotton, Hristina Dimitrova, Thomas Hickman, Michael Norman
- **UCLouvain:** Guillaume Cerckel, Alexandre Jolly, Benjamin Ledoux, Marine Lefevre, Cyril Marechal, Pierre Van Meerbeeck, Caroline de Pret, Thomas Reginster, Roxane Schleusner, Olivier Suys
- **UConn:** Juliana Crivello, Alessandro Fisher, Brittany Galuppo, Nicholas Hellmann, Juan Jeffrey, Sricharan Kadimi, Kendra Maas, Craig Mendonca, Rachel O'Neil, Charlie Wright
- **UCopenhagen:** Jon Fugl, Cecilie Hansen, Stine Tougaard
- **UCSC:** Walter Bray, Scott Lokey, Alexander Pearce, Evan Pepper, Daniel Schmelter, Thomas Sousa, Brittney Wick
- **UFlorida:** Melanie Correll, Erin Guby, Shane Kammerman, Hannah Schmidt, Madeline Steck
- **UGA-Georgia:** Bidushi Chandra, Kyler Herrington, Esther Kim, Chynna Pollitt
- **UiOslo_Norway:** Daniel Hatlem, Dirk Linke, Dejana Mitrovic, Eric de Muinck, Kristina Randjelovic, Athanasios Saragliadis, Elena Stojanovska, Hallvard Storkaas, Eirik Bager Sundmark, Can Hicabi Tartanoglu
- **UIOWA:** Henry J Conlan, James Cory, Craig Ellermeier, Jan Fassler, Mason Lamarche, Nicholas S McCarty, Paige Noble, Edward Sander, Paul V Taufalele, Nina VanDerZanden
- **UIUC_Illinois:** Ayesha Ellahi, Qingxi Meng, Nathan Suryajaya Purwanto
- **UMaryland:** Vaidehi Bhagat, Narendranath Bhokisham, Seth Cohen, Cameron Harner, Paula Kleyman, Asha Kodan, Aleksandr Kovalyonok, Chun Mun Loke, Thea Orstein, Jacob Premo, Yuzhu Shi, Joyce Song, Annie Trang
- **UNBC-Canada:** Jason Chu, Brendan Reiter
- **UNC-Asheville:** Katie Brown, William Jackson, Nick White
- **UNebraska-Lincoln:** Nicholas Flaxbeard, Nicholas Kite, Wei Niu
- **UNOTT:** Eleanor Boardman, Ines Canadas, Matt French, Chris L.B. Graham, Daphne Groothuis, Natalia Kotynska, Georgette Sebastio, Jake Yeboah, Maria Zygouropoulou
- **Uppsala:** Lucie H. Kulhankova, Friederike Mey, Teresa Reinli, Sai Sreekar Wunnava Venkata

- **UrbanTundra_Edmonton:** Ejouan Agena, Rochelin Dalangin, Jing Huang, Caleb Loo, Tracy Vuong, Andrew Wu, Derrick Zhang
- **US_AFRL_CarrollHS:** Anna Bennet, Annie Bete, Tina Davis, Jason Dong, Svetlana Harbaugh, Haylee Jesse, Dallas McDonald
- **USMA-West_Point:** Zach Andersen, Alana Appel, John Cave, Jason Hug, Elizabeth Huuki, Kylor Kiesewetter, Dillon Macky, Matthew McDonough, Channah Mills, Alex Mitropoulos, James Pruneski, J. Ken Wickiser, Kamil Woronowicz
- **USNA_Annapolis:** Natalia Barrow, Dwayne Williams
- **USP-Brazil:** Tiago Lubiana Alves, Felipe Xavier Buson, Gabriel Lencioni Lovate, Caua Antunes Westmann
- **UST_Beijing:** Zhang Chi, Zhong Jinjin, Liu Peiyuan, Xu Qi, Guo Shuning, Zhang Shuyuan, Ching Song, Xu Suiping, Xue Tiantian, Guo Yannan, Zhang Yixuan, Peng Jiarong Wei Yuting, Hang Zhongci
- **UT-Knoxville:** Jared Clements, Ralph Laurel, Jay Patel, James Ragland, Elle Ridley, Thomas Welker
- **Utrecht:** Pamela Capendale, Lishi Lin, Glenn Mulder, Kewin Ogink, Giel Scheepers
- **Vilnius-Lithuania:** Aukse Gaizauskaite, Laurynas Karpus, Ignas Mazelis, Justas Ritmejeris, Irmantas Rokaitis, Antanas Zilakauskis
- **Virginia:** Ilya Andreev, Kay Christopher, Keith G Kozminski, Steven Scherping, Eric Joshua Wang
- **Wageningen_UR:** Stijn Prinsen, Jurre Steens
- **Warwick:** Clare Hayes, Elsita Jungkurth, Jack Lawrence, Amy Moore, Robert Richardson, Balint Vidos
- **Washington:** Karl Anderson, Erin Broderick, Alan Cabrera, Emily Chun, William Kwok, Kara de Leon, Kevin Li, Texia Loh, Michael Nguyen, Cameron Roots, Angel Wong
- **WashU_StLouis:** Collin Kilgore, Maddie Lee, Zoe Orenstein, Micah Rickles-Young, Mark Wang, Alex Yenkin
- **WHU-China:** Yu Chen, Yuxuan Chen, Guangyuan Li, Xiaohui Xue, Jiawei Yu, Jing Zhang, Xinru Zhang, Luyang Zhao
- **William_and_Mary:** Sejal Dhawan, Theresa Gibney, Ethan Jones, Christine Li, Wukun Liu, Alyssa Luz-Rica, John Marken, Callan Monette, Xida Ren, Margret Saha, Greg Smith, Xingyu Zheng
- **Worldshaper-Nanjing:** Sam Dong
- **WPI_Worcester:** Locke Bonomo, Natalie Farny, Haylea Northcott, Aylin Padir, Michael Savoie, Edith Sawyer, Catherine Sherman
- **XJTLU-CHINA:** Ziyin Jin, Yiyou Song, Tianjun Wang
- **XMU-China:** Yousi Fu, Yulong Han, Yang Liang
- **ZJU-China:** Shisheng Li, Cheng Shen, Zifan Xie, Haiyan Yan
- **ZJUT-China:** Geyi Wang, Yisha Zhu

2018 Teams

The iGEM 2018 Interlab Contributors comprise a total of 1399 authors from 244 institutions.

- **Aachen:** Meryem Pehlivan, Biel Badia Roige
- **Aalto-Helsinki:** Tiu Aarnio, Samu Kivisto, Jessica Koski, Leevi Lehtonen, Denise Pezzutto, Pauliina Rautanen
- **AHUT_China:** Weixin Bian, Zhiyuan Hu, Zhihao Liu, Zi Liu, Liang Ma, Luyao Pan, Zichen Qin, Huichao Wang, Xiangxuan Wang, Hao Xu, Xia Xu
- **Aix-Marseille:** Yorgo El Moubayed
- **ASTWS-China:** Shan Dong, Choco Fang, Hanker He, Henry He, Fangliang Huang, Ruyi Shi, Cassie Tang, Christian Tang, Shirly Xu, Calvin Yan
- **Athens:** Natalia Bartzoka, Eleni Kanata, Maria Kapsokefalou, Xanthi-Leda Katopodi, Eleni Kostadima, Ioannis V. Kostopoulos, Stylianos Kotzastratis, Antonios E. Koutelidakis, Vasilios Krokos, Maria Litsa, Ioannis Ntekas, Panagiotis Spatharas, Ourania E. Tsitsilonis, Anastasia Zerva
- **Austin_LASA:** Vidhya Annem, Eli Cone, Noel Elias, Shreya Gupta, Kendrick Lam, Anna Tutuianu
- **Austin_UTexas:** Dennis M. Mishler, Bibiana Toro
- **Baltimore_BioCrew:** Akinwumi Akinfenwa, Frank Burns, Heydy Herbert, Melissa Jones, Sarah Laun, Shikei Morrison, Zion Smith
- **BCU:** Zhao Peng, Zhou Ziwei
- **BFSUICC-China:** Rui Deng, Yilin Huang, Tingyue Li, Yingqi Ma, Zhiyuan Shen, Chenxi Wang, Yuyao Wang, Tianyan Zhao
- **BGIC-Global:** Yusen Lang, Yuteng Liang, Xueyao Wang, Yi Wu
- **BGU_Israel:** Dror Aizik, Sagi Angel, Einan Farhi, Nitzan Keidar, Eden Oser, Mor Pasi
- **Bielefeld-CeBiTec:** Jorn Kalinowski, Matthias Otto, Johannes Ruhnau
- **Bilkent-UNAMBG:** Hande Cubukcu, Mehmet Ali Hoskan, Ilayda Senyuz
- **BioIQS-Barcelona:** Jordi Chi, Antoni Planas Sauter, Magda Faijes Simona
- **BioMarvel:** Sumin Byun, Sungwoo Cho, Goeun Kim, Yeonjae Lee, Sangwu Lim, Hanyeol Yang
- **BIT:** Tian Xin, Zhang Yaxi, Peng Zhao
- **BIT-China:** Weitang Han, Fa He, Yuna He, Nuonan Li, Xiaofan Luo
- **BJRS_China:** Cheng Boxuan, Hu Jiaqi, Yang Liangjian, Li Wanji, Chen Xinguang, Liu Xinyu
- **BNDS_CHINA:** Zishi Wu, Yukun Xi, Xilin Yang, Yuchen Yang, Zhuoyi Yang, Yihao Zhang, Yuezhang Zhou
- **BNU-China:** Yue Peng, Liu Yadi, Shaobo Yang, Jiang Yuanxu, Kecheng Zhang
- **BOKU-Vienna:** Doris Abraham, Theresa Heger
- **BostonU:** Cass Leach, Kevin Lorch, Linda Luo

- **British_Columbia:** Alex Gaudi, Anthony Ho, Morris Huang, Christine Kim, Luxcia Kugathasan, Kevin Lam, Catherine Pan, Ariel Qi, Cathy Yan
- **Calgary:** Kaitlin Schaaf, Cassandra Sillner
- **Cardiff_Wales:** Ryan Coates, Hannah Elliott, Emily Heath, Evie McShane, Geraint Parry, Ali Tariq, Sophie Thomas
- **CCU_Taiwan:** Ching-Wei Chen, Yu-Hong Cheng, Chia-Wei Hsu, Chin-Hsuan Liao, Wei-Ting Liu, Yu-Cheng Tang, Yu-Hsin Tang, Zon En Yang
- **CDHSU-CHINA:** Liu Jian, Caidian Li, Chenyi Lin, Guozheng Ran, Zhouyan Run, Weiyu Ting, Zhangxiang Yong, Liuhong Yu
- **Chalmers-Gothenburg:** Andrea Clausen Lind, Axel Norberg, Amanda Olmin, Jacob Sjolin, Agnes Torell, Cecilia Trivellin, Francisco Zorrilla, Philip Gorter de Vries
- **CIEI-BJ:** Haolun Cheng, Jiarong Peng, Zhenyu Xiong
- **CMUQ:** Dina Altarawneh, Sayeda Sakina Amir, Sondoss Hassan, Annette Vincent
- **CO_Mines:** Ben Costa, Isabella Gallegos, Mitch Hale, Matt Sonnier, Kathleen Whalen
- **ColumbiaNYC:** Max Elikan, Sean Kim, Jaewon You
- **Cornell:** Rahul Rambhatla, Ashwin Viswanathan
- **CPU_CHINA:** Hong Tian, Huandi Xu, Wanli Zhang, Shuyao Zhou
- **CSU_CHINA:** Liu Jiamiao, Xiao Jiaqi
- **CSU_Fort_Collins:** Darilyn Craw, Marley Goetz, Neil Rettedal, Hayden Yarbrough
- **Delgado-Ivy-Marin:** Christopher Ahlgren, Brett Guadagnino, James Guenther, Juilanne Huynh
- **DLUT_China:** Zhien He, Huan Liu, Yuansheng Liu, Mingbo Qu, Li Song, Chao Yang, Jun Yang, Xianqi Yin, Yuanzhen Zhang, Jianan Zhou, Lihan Zi
- **DLUT_China_B:** Zhu Jinyu, Xu Kang, Peng Xilei, Han Xue, Shu Xun
- **DNHS_SanDiego:** Priyanka Babu, Arushi Dogra, Pranav Thokachichu
- **DTU-Denmark:** David Faurdal, Joen Haahr Jensen, Jacob Mejlsted, Lina Nielsen, Tenna Rasmussen
- **Duesseldorf:** Jennifer Denter, Kai Husnatter, Ylenia Longo
- **Ecuador:** Juan Carlos Luzuriaga, Eduardo Moncayo, Natalia Torres Moreira, Jennifer Tapia
- **ECUST:** Tang Dingyue, Zhao Jingjing, Xu Wenhao, Teng Xinyu, Hong Xiujing
- **Edinburgh_OG:** Jackson DeKloe
- **Edinburgh_UG:** Ben Astles, Ugne Baronaite, Inga Grazulyte
- **Emory:** Michael Hwang, Yibo Pang
- **EPFL:** Michael Andrew Crone, Reza Hosseini, Moustafa Houmani, Daniel Zadeh, Violetta Zanotti
- **ETH_Zurich:** Oliver Andreas Baltensperger, Eline Yafele Bijman, Elisa Garulli, Jan Lukas Krusemann, Adriano Martinelli, Antonio Martinez, Tobias Vornholt

- **Evry_Paris-Saclay:** Monteil Camille, Ahavi Paul
- **Exeter:** Emily Browne, Daniel Barber James Gilman, Amy Hewitt, Sophie Hodson, Ingebjorg Holmedal, Fiona Kennedy, Juliana Sackey
- **FAU_Erlangen:** Selina Beck, Franziska Eidloth, Markus Imgold, Anna Matheis, Tanja Meerbrei, David Ruscher, Marco Schaeftlein
- **FJNU-China:** Zhu Hanrong
- **Fudan:** Mitchell Wan
- **Fudan-CHINA:** Leijie Dai, Kaifeng Jin, Sihan Wang, Xin Wang, Yi Wang, Yifan Wang, Chenhai Wu, Zixuan Zhang, Yineng Zhou
- **GDSYZX:** Liu Xinyu, Zeng Zirong
- **Georgia_State:** Rehmat Babar, Mathew Brewer, Christina Clodomir, Laura Das Neves, Amanda Iwuogo, Ari Jones, Cara Jones, Julia Kelly, Gloria Kim, Jessica Siemer, Yash Yadav
- **Gifu:** Yuichiro Ikagawa, Tatsuki Isogai, Ryo Niwa
- **GO_Paris-Saclay:** Celine Aubry, William Briand, Annick Jacq, Sylvie Lautru, Britany Marta, Clemence Maupu, Xavier Ollessa-Daragon, Kenn Papadopoulo, Mahnaz Sabeta Azad
- **GreatBay_China:** Wei Kuangyi, Yao Xiu, Chenghao Yang
- **Groningen:** Aditya Iyer, Rianne Prins, Phillip Yesley
- **GZHS-United:** Fang Lichi, Chen Zi Xuan
- **HAFS:** Kyuhee Jo, Mikyung Park, Seunghyun Park, Hojun Yoo
- **Hamburg:** Nele Burckhardt, Lea Daniels, Bjarne Klopprogge, Dustin Kruger, Oda-Emilia Meyfarth, Lisa Putthoff, Dominika Wawrzyniak
- **HBUT-China:** Xinyi Hu, Yunyi Wang
- **HebrewU:** Lior Badash, Amichai Baichman-Kass, Alon Barshap, Yonatan Friedman, Eliya Milshtein, Omri Vardi
- **HFLS_ZhejiangUnited:** Shan Dong, Yining Gu, Yuanzhe Pei, Ruyi Shi, Fan Yang, Jinshu Yang, Xueqian Zhu
- **HK_HCY_LFC:** Lam Kai Ching, Law Hiu Ching, Ng Tsz Chun, Yu Man Hin, Lai Tsz Hong, Chan Wing Lam, Yiu Choi Lam, Cheah Matthew, Cheng Tsz Ngo, Yun Shuan, Chan Tsey Wan, Tsui Shing Yan, Chong Yuk Yee, Tam Chi Yu, Yuen Wai Yu
- **HKJS_S:** Chung Tsun Ho Anson, Lee Sze Choi, Cheung Man Chun, Chan Lok Hin, Wong Chung Hin, Ng Sze Ho, Leung Chung Yin Jay, Lai Man Wai Katherine, Wong Carol Kin-ning, Lee Hong Kiu, Cheng Chak Kong, Leung Chung Wai, Yeung Wing Yan, Wong Tsz Yeung, Lee Ka Yin
- **Hong_Kong_HKU:** Tsui Shing Yan Grace, Lam Kai Ching Joe, Ng Tsz Chun Kenneth, Cheah Matthew Yun Shuan
- **Hong_Kong_HKUST:** Ferdinan Aldo, Chung Him Pang, Kam Pang So, Hei Man Wong
- **Hong_Kong_JSS:** Lai Tsz Ching, Luk Hau Ching, Ip Ning Fung, Yam Shing Fung, Lee Chi Hong, Hsiu Ou Ning, Jonathan Cheng Hon Sang

- **Hong_Kong-CUHK:** Yeung Hoi Lam Elsa, Chan Yick Hei, Lo Ho Sing, Choi Seong Wang
- **HUBU-Wuhan:** Yiheng Gu, Ziyue Rong, Haoyue Song, Pengying Wang, Yuefei Wang
- **HUST-China:** Yan Chen, Hao Qiu, Haotian Ren, Ziyang Xiao
- **HZAU-China:** Heng Heng, Xichen Rao, Ruonan Tian
- **ICT-Mumbai:** Shalini S. Deb, Yash Laxman Kamble, Ninad Kumbhojkar, Marwan Malik, Bhargav Patel, Supriya Prakash, Shamlan M.S. Reshamwala, Poorva Taskar
- **IISc-Bangalore:** Gokul, Adwaith B Uday
- **IISER-Bhopal-India:** Anubhav Basu, Rishi Gandhi, Jatin Khaimani, Arundhati Khenwar, Sandeep Raut, Tejas Somvanshi
- **IISER-Kolkata:** Diptatanu Das, Souvik Ghosh, Hrishika Rai
- **IISER-Mohali:** Nithishwer Mouroug Anand, Ashwin Kumar Jainarayanan, Pranshu Kalson, Devang Haresh Liya, Vibhu Mishra, Sveekruth Sheshagiri Pai, Madhav Pitaliya, Yash Rana, Ravineet Yadav
- **IIT_Delhi:** Neha Arora, Vasu Arora, Shubham Jain, Abhilash Patel, Saksham Sharma, Priyanka Singh
- **IIT_Kanpur:** Anushya Goenka, Rishabh Jain, Aryaman Jha, Adarsh Kumar, Abhinav Soni
- **IIT-Madras:** Sathvik Ananthakrishnan, Velvizhi Devi, Mohammed Faidh, Guhan Jayaraman, M Sagar Kittur, Nitish R Mahapatra, Sarvesh Menon, Anantha Barathi Muthukrishnan, Kailash B P, Burhanuddin Sabuwala, Mousami Shinde, Sankalpa Venkatraghavan
- **Jiangnan_China:** Weijia Liu, Zhoudi Miao, Tian Wang, Yaling Wang, Shuyan Zhang
- **Jilin_China:** Ruochen Chai, Yubin Ge, Ali Hou, Fangqi Liu, Xutong Liu, Jiangjiao Mao, Zihao Wang, Haimeng Yu, Hetian Yuan, Yang Zhan
- **JMU_Wuerzburg:** Anna Ries, Chiara Wolfbeisz
- **KAIT_JAPAN:** Toshihiro Kanaya, Yusuke Kawasaki, Tatuya Maruo, Yuya Mori, Takehito Satoh
- **KCL_UK:** Anthony Chau, Wai Yan Chu, Anatoliy Markiv, Marcos Vega-Hazas Marti, Maria Jose Ramos Medina, Deeksha Raju, Shubhankar Sinha
- **KUAS_Korea:** Youngeun Choi, Bo Sun Ryu
- **Lambert_GA:** Gaurav Byagathvalli, Ellie Kim
- **Leiden:** Marjolein Crooijmans, Jazzy de Waard, Chiel van Amstel
- **Lethbridge:** Aubrey Demchuk, Travis Haight, Dong Ju Kim, Andrei Neda, Luc Roberts, Luke Saville, Reanna Takeyasu, David Tobin
- **Lethbridge_HS:** Mina Akbary, Rebecca Avileli, Karen He, Aroma Pageni, Luke Saville, Dewuni De Silva, Nimaya De Silva, Kristi Turton, Michelle Wu, Alice Zhang
- **Lubbock_TTU:** Benjamin Chavez, Paula Garavito, Michael Latham, Jeffrey Ptak, Darron Tharp
- **Lund:** Nurul Izzati, Martin Jonsson, Nikol Labecka, Sara Palo
- **Macquarie_Australia:** Renee Beale, Dominic Logel, Areti-Efremia Mellou, Karl Myers

- **Madrid-OLM:** Alejandro Alonso, Rodrigo Hernandez Cifuentes, Borja Sanchez Clemente, Gonzalo Saiz Gonzalo, Ivan Martin Hernandez, Laura Armero Hernandez, Francisco Javier Quero Lombardero, Domingo Marquina, Guillermo Fernandez Rodriguez, Ignacio Albert Smet
- **Manchester:** Tom Butterfield, Ed Deshmukh-Reeves, Namrata Gogineni, Sam Hemmings, Ismat Kabbara, Ieva Norvaisaite, Ryan Smith
- **Marburg:** Daniel Bauersachs, Benjamin Daniel, Rene Inckemann, Alexandra Seiffermann, Daniel Stukenberg, Carl Weile
- **McGill:** Valerian Clerc, Jacqueline Ha, Stephanie Totten
- **McMaster:** Thomas Chang, Carlene Jimenez, Dhanyasri Maddiboina
- **METU_HS_Ankara:** Beliz Leyla Acar, Evrim Elcin, Tugba Inanc, Gamze Kantas, Ceyhun Kayihan, Mert Secen, Gun Suer, Kutay Ucan, Tunc Unal
- **Michigan:** Matthew Fischer, Naveen Jasti, Thomas Stewart
- **MichiganState:** Sarah Caldwell, Jordan Lee, Jessica Schultz
- **Mingdao:** Ting-Chen Chang, Pei-Hong Chen, Yu-Hsuan Cheng, Yi-Hsuan Hsu, Chan-yu Yeh
- **Minnesota:** Zhipeng Ding, Zihao Li, Savannah Lockwood, Katherine Quinn
- **Montpellier:** Leo Carrillo, Maxime Heintze, Lea Meneu, Marie Peras, Tamara Yehouessi
- **Munich:** Keno Eilers, Elisabeth Falgenhauer, Wong Hoi Kiu, Julia Mayer, Julia Mueller, Sophie von Schoenberg, Dominic Schwarz, Brigit Tunaj
- **Nanjing-China:** Zhaoqing Hu, Yansong Huang, Yuanyuan Li
- **NAU-CHINA:** Chengzhu Fang, Jiangyuan Liu, Yiheng Liu, Yaxuan Wu, Sheng Xu, Long Yuan
- **NAWI_Graz:** Marco Edelmayer, Marlene Hiesinger, Sebastian Hofer, Birgit Krainer, Andreas Oswald, Dominik Strasser, Andreas Zimmermann
- **NCHU_Taichung:** Yi-Cian Chen
- **NCTU_Formosa:** Yuan-Yao Chan, Yu-Ci Chang, Nian Ruei Deng, Chi-Yao Ku, Meng-Zhan Lee
- **NEU_China_A:** Hailong Li, Zhaoyu Liu, Guowei Song, Yuening Xiang, Hongfa Yan
- **NEU_China_B:** He Huanying, Jiang Qiaochu, Jiang Shengjuan, Peng Yujie
- **Newcastle:** Matt Burridge, Kyle Stanforth, Sam Went
- **NJU-China:** Liang Chenxi, Wang Han, Zhang Qipeng, Li Yifan, Quan Yiming, Pan Yutong
- **NKU_CHINA:** Senhao Kou, Lin Luan
- **Northwestern:** Umut Akova, Liza Fitzgerald, Bon Ikwuagwu, Michael Johnson, Jacob Kurian, Christian Throsberg
- **Nottingham:** Lucy Allen, Christopher Humphreys, Daniel Partridge, Michaella Whittle, Nemira Zilinskaite
- **NPU-China:** Meixuan Lee, Weifeng Lin, Yuan Ma, Kai Wang
- **NTHU_Formosa:** Hsuan Cheng, Shumei Chi, Yi-Chien Chuang, Ray Huang, LiangYu Ko, Yu-Chun Lin

- **NTHU_Taiwan:** You-Yang Tsai, Cheng-Chieh Wang, Kai-Chiang Yu
- **NTNU_Trondheim:** Hanna Nedreberg Burud, Carmen Chen, Anne Kristin Haralsvik, Adrian Marinovic, Hege Hetland Pedersen, Amanda Sande, Vanessa Solvang
- **NTU-Singapore:** Shaw Kar Ming, Albert Praditya
- **NU_Kazakhstan:** Aiganym Abduraimova, Ayagoz Meirkhanova, Assel Mukhanova, Tomiris Mulikova
- **NUDT_CHINA:** Yanchen Gou, Chenyu Lu, Jiaxin Ma, Chushu Zhu
- **NUS_Singapore-A:** Leow Chung Yong Aaron, Tvarita Shivakumar Iyer, Wu Jiacheng, Yan Ping Lim, Beatrix Tung Xue Lin, Aaron Ramzeen, Nur Liyana Binte Ayub Ow Yong
- **NUS_Singapore-Sci:** Yah Tse Sabrina Chua, Yuhui Deborah Fong, Menglan He, Li Yang Tan
- **NWU-China:** Zhang Jiahe, Li Mingge, Li Nianlong, Li Yueyi, Cheng Yuhan
- **NYMU-Taipei:** Annabel Chang, Chih-Chiang Chen, Ryan Chou, Jude Clapper, Evelyn Lai, Yasmin Lin, Kelsey Wang, Jake Yang
- **NYU_Abu_Dhabi:** Mariam Anwar, Ibrahim Chehade, Imtiyaz Hariyani, Sion Hau, Ashley Isaac, Laura Karpauskaite, Mazin Magzoub, Daniel Obaji, Yong Rafael Song, Yejie Yun
- **OUC-China:** Kai Sun, Yunqian Zhang
- **Oxford:** Eleanor Beard, Laurel Constanti Crosby, Nicolas Delalez, Arman Karshenas, Adrian Kozhevnikov, Jhanna Kryukova, Karandip Saini, Jon Stocks, Bhuvana Sudarshan, Max Taylor, George Wadhams, Joe Windo
- **Paris_Bettencourt:** Annissa Ameziane, Darshak Bhatt, Alexis Casas, Antoine Levrier, Ana Santos, Nympha Elisa M. Sia, Edwin Wintermute
- **Pasteur_Paris:** Alice Dejoux, Deshmukh Gopaul, Lea Guerassimoff, Samuel Jaoui, Manon Madelenat, Serena Petracchini
- **Peking:** Fu Cai, Yang Jianzhao, Shi Shuyu, Li Tairan, Li Xin, Lin Yongjie, Huang Zhecheng
- **Pittsburgh:** Evan Becker, Matthew Greenwald, Vivian Hu, Tucker Pavelek, Elizabeth Pinto, Zemeng Wei
- **Purdue:** Zachary Burgland, Janice Chan, Julianne Dejoie, Kevin Fitzgerald, Zach Hartley, Moiz Rasheed, Makayla Schacht
- **Queens_Canada:** Maddison Gahagan, Ellis Kelly, Elisha Krauss
- **RDFZ-China:** Yuze Cao, Yishen Shen, Xuan Wang, Hanning Xu, Jianxiang Zhang
- **REC-CHENNAI:** Priyanka Chandramouli, Amal Jude Ashwin F, Srimathi Jayaraman, Marcia Smiti Jude, Vignesh Kumar, Hema Lekshmi, Preetha R, Khadija Rashid, Deepak Kumar S, Mohan Kumar B S
- **Rheda_Bielefeld:** Leon Michael Barrat, Jil-Sophie Dissmann, Jorn Kalinowski, Matthias Otto, Johannes Ruhnau, Fynn Stuhlweissenburg, Elisa Ueding
- **RHIT:** Ariel Bohner, Brittany Clark, Emilie Deibel, Liz Klaas, Kaylee Pate, Elisa Weber
- **Rice:** Katherine Cohen, Anna Guseva, Stefanie King, Soohyun Yoon

- **Ruia-Mumbai:** Sanika Ambre, Shilpa Bhowmick, Nishtha Pange, Komal Parab, Vainav Patel, Mitali Patil, Aishwarya Rajurkar, Mayuri Rege, Maithili Sawant, Shrutika Sawant, Anjali Vaidya
- **SBS_SH_112144:** Peicheng Ji, Fang Luo, Guanghui Ma, Xin Xu, Jiacheng Yin, Yinchi Zhou, Ke Zhu
- **SCAU-China:** Yaohua Huang, Yinpin Huang, Jiadong Li, Xuecheng Li, Hao Wang, Ken Wang, Wei Wang, Xinyu Zhang, Jiahua Zou
- **SCU-China:** Minyue Bao, Han Kang, Xiaolong Liu, Yibing Tao, Ziru Wang, Fuqiang Yang, Tianyi Zhang, Yanling Zhong
- **SCUT_ChinaB:** Jiezheng Liu, Jingang Liu, Lingling Ma, Xubo Niu, Ling Qian, Li Wang, Qingyan Yan, Nannan Zhao
- **SCUT-ChinaA:** Weixuan Chen, Yuxin Zhou
- **SDU-CHINA:** Junyang Chen
- **SFLS_Shenzhen:** Junyao Hao, Zhang HuaYue, Peilin Li, Yifei Pei, Jingting Qu, Raven Wang, Xinyue Wang, Kangjie Wu, Yuxuan Wu, Meredith Xiang, Leyi Yang, Zisang Yang, Li Zhaoting
- **ShanghaiTech:** Wenhan Fu, Zonghao Li, Weiyi Tang, Kaida Zhang
- **SHSBNU_China:** Haocong Li, Xuze Shao, Chuyi Yang, Yuanhong Zeng, Yanjun Zhou
- **SHSID_China:** Shangzhi Dong, Younji Jung, Sophie Ruojia Li, Tingting Li, Jiacheng Yu
- **SHSU_China:** Shangzhi Dong, Tingting Li, Xinyi Miao, Sibo Wang
- **SIAT-SCIE:** Yiming Ding, Jiaxi Huang, Yuqi Li, Ting Sun, Qinghe Tian, Mengxuan Wu, Jinming Xing, Xin Xiong, Yining Yan, Qiu Yihang, Jige Zhang, Yi Zhou, Zhiyu Zhou
- **SJTU-BioX-Shanghai:** Zhuoyang Chen, Peixiang He, Yirui Hong, Chia-Yi Hsiao, Zhihan Liang, Zhixiang Liu, Yuncong Ran, Shiyu Sun, Ruoyu Xia
- **SKLMT-China:** Dongyang Dong, Wenxue Zhao
- **SMMU-China:** Miao Hu, Shi Hu, Wei Shi, Shulun, Han Yan, Yusheng Ye
- **SMS_Shenzhen:** Yiquan Hong, Yuyao Pan, Yiran Song, Jinhan Zhang, Yihang Zhao
- **Sorbonne_U_Paris:** Dounia Chater, Asmaa Foda, Yanyan Li, Ursula Saade, Victor Sayous
- **SSHS-Shenzhen:** Yilin Mo, Wenan Ren, Chenxu Zeng
- **SSTi-SZGD:** Yixin Cao
- **St_Andrews:** Clarissa Czekster, Izzy Dunstan, Simon Powis, Bethany Reaney, Eva Snaith, Cam Young
- **Stanford:** Eva Frankel, Eleanor Glockner, Isaac Justice
- **Stanford-Brown-RISD:** Santosh Murugan, Leo Penny
- **Stockholm:** Chrismar Garcia, Stamatina Rentouli
- **Stony_Brook:** Priya Aggarwal, Stephanie Budhan, Woody Chiang, Dominika Kwasniak, Karthik Ledalla, Matthew Lee, Natalie Lo, Matthew Mullin, Lin Yu Pan, Jennifer Rakhimov, Robert Ruzic, Manvi Shah, Lukas Velikov, Sara Vincent

- **Stuttgart:** Philip Horz, Nadine Kuebler, Jan Notheisen
- **SUIS_Shanghai:** David Doyle, Jiajun Gu, Wenyue Hu, Shuting Yang
- **SYSU-CHINA:** Tao Kehan, Gao Menghan, Mao Xiaowen
- **SYSU-Software:** Yifei Chen, Ziqi Kang, Haochen Ni
- **SZU-China:** Junyu Chen, Lindong He, Mingyue Luo, Jiaqi Tang
- **Tacoma_RAINmakers:** Kira Boyce, James Lee, Michael Martin, Judy Van Nguyen, Leon Wan
- **Tartu_TUIT:** Artur Astapenka, Turan Badalli, Irina Borovko, Nadezhda Chulkova, Ilona Faustova, Anastasia Kolosova, Mart Loog, Artemi Maljavin, Frida Matiyevskaya, Vladislav Tuzov
- **TAS_Taipei:** Catherine Chang, Ryan Chou, Jude Clapper, Tim Ho, Yi Da Hsieh, Evelyn Lai, Leona Tsai, Kelsey Wang, Justin Wu
- **Tec-Chihuahua:** Viana Isabel Perez Dominguez, Cesar Ibrahym Rodriguez Fernandez, Daniela Olono Fierro, Anna Karen Aguilar Nunez, Jose Pablo Rascon Perez, Mario Loya Rivera, Cynthia Lizeth Gonzalez Trevizo, Maria Antonia Luna Velasco
- **Tec-Monterrey:** Carlos Javier Cordero Oropeza, Adrian Federico Hernandez Mendoza, Jose Arnulfo Juarez Figueroa, Luis Mario Leal, Samantha Ayde Pena Benavides, Victor Javier Robledo Martinez, Adriana Lizeth Rubio Aguirre, Andres Benjamin Sanchez Alvarado, Margarita Sofia Calixto Solano, Nora Esther Torres Castillo, Alejandro Robles Zamora, Esteban de la Pena Thevenet
- **TecCEM:** Karla Soto Blas, Ana Laura Torres Huerta, Armando Cortes Resendiz
- **TecMonterrey_GDL:** Frida Cruz, Fernanda Diaz, Diego Espinoza, Ana Cristina Figueroa, Ana Cecilia Luque, Roberto Portillo, Carolina Senes, Diana Tamayo, Mariano Del Toro
- **Thessaloniki:** Ioannis Alexopoulos, Alexandros Dimitriou Giannopoulos, Yvoni Giannoula, Grigorios Kyrpizidis
- **Tongji_China:** Ma Xinyue, Chen Xirui, Song Zhiwei
- **Toronto:** Nina Adler, Amalia Caballero, Carla Hamady, Ahmed Ibrahim, Jasmeen Parmar, Tashi Rastogi, Jindian Yang
- **Toulouse-INSA-UPS:** Jean Delhomme, Anthony Henras, Stephanie Heux, Yves Romeo, Marion Toanen, Camille Wagner, Paul Zanoni
- **TU_Darmstadt:** Thea Lotz, Elena Nickels, Beatrix Suss, Heribert Warzecha, Jennifer Zimmermann
- **TU-Eindhoven:** Emilien Dubuc, Bruno Eijkens, Sander Keij, Simone Twisk, Mick Verhagen, Maxime van den Oetelaar
- **TUDelft:** Alexander Armstrong, Nicole Bennis, Susan Bouwmeester, Lisa Buller, Kavish Kohabir, Monique de Leeuw, Venda Mangkusaputra, Jard Mattens, Janine Nijenhuis, Timmy Paez, Lisbeth Schmidtchen, Gemma van der Voort
- **TUST_China:** Gao Ge, Xu Haoran, Li Xiaojin
- **UAlberta:** Ejouan Agena, Ethan Agena, Scott Bath, Robert Campbell, Rochelin Dalangin, Anna Kim, Dominic Sauvageau, Irene Shkolnikov
- **UC_Davis:** Daniel Graves, Jacob Lang, Jolee Nieberding-Swanberg, Achala Rao, Ares Torres, Andrew Yao

- **UC_San_Diego:** Anser Abbas, Claire Luo
- **UCAS-China:** Xu Zepeng, Zhao Ziyi
- **UChicago:** Janice Chen, Cian Colgan, Steve Dvorkin, Rachael Filzen, Varun Patel, Allison Scott, Patricia Zulueta
- **UChile_Biotec:** Joaquin Acosta, Lucas Araya, Francisco Chavez, Sebastian Farias, Delia Garrido, Andres Marcoleta, Felipe Munoz, Paula Rivas
- **UCL:** Noelle Colant, Catherine Fan, Stefanie Frank, Jacopo Gabrielli, Paola Handal, Vitor Pinheiro, Stefanie Santamaria, Shamal Withanage, Fang Xue
- **UCLouvain:** Antoine Gerard, Marine Lefevre, Fiona Milano, Nina De Sousa Oliveira, Mathieu Parmentier, Luca Rigon
- **UConn:** Elizabeth Chamiec-Case, Ryan Chen, Peter Crowley, Shannon Doyle, Sricharan Kadimi, Toni Vella
- **UCopenhagen:** Natthawut Adulyanukosol, Theodore A Dusseaux, Victor Forman, Cecilie Hansen, Selma Kofoed, Simon Louis, Magnus Ronne Lykkegaard, Davide Mancinotti, Lasse Meyer, Stephanie Michelsen, Morten Raadam, Victoria Svaerke Rasmussen, Eirikur Andri Thormar, Attila Uslu, Nattawut leelahakorn
- **UESTC-China:** Shizhi Ding, Changyu Li, Huishuang Tan, Yinsong Xu, Jianzhe Yang
- **UFlorida:** Diego Gamoneda, Nicole Kantor, Lidimarie Trujillo-Rodriguez, Matthew Turner
- **UGA:** Stephan George, Kelton McConnell, Chynna Pollitt
- **UI_Indonesia:** Ihya Fakhrurizal Amin, Muhammad Ikhsan, Valdi Ven Japranata, Andrea Laurentius, Luthfian Aby Nurachman, Muhammad Iqbal Adi Pratama
- **UiOslo_Norway:** Yvette Dirven, Lisa Frohlich, Dirk Linke, Verena Mertes, Rebekka Rekkedal Rolfsnes, Athanasios Saragliadis
- **UIOWA:** Sandra Castillo, Sathivel Chinnathambi, Craig Ellermeier, Jennifer Farrell, Jan Fassler, Ernie Fuentes, Sean Ryan, Edward Sander
- **UIUC_Illinois:** Amie Bott, Liam Healy, Pranathi Karumanchi, Alex Ruzicka, Ziyu Wang
- **ULaval:** Gabriel Byatt, Philippe C Despres, Alexandre Dube, Florian Echelard, Pascale Lemieux, Louis-Andre Lortie, Francois D Rouleau
- **ULaVerne_Collab:** Seth Barrington, Cynthia Basulto, Sabrina Delgadillo, Karen De Leon, Micah Madrid, Catherosette Meas, Angelica Sabandal, Magaly Aguirre Sanchez, Jennifer Tsui, Noble Woodward
- **UMaryland:** Rohith Battina, Jess Boyer, Arjun Cherupalla, Jason Chiang, Mary Heng, Collin Keating, Tommy Liang, Chun Kit Loke, Jacob Premo, Keerthana Srinivasan, John Starkel, Daniel Zheng
- **UNebraska-Lincoln:** Gabe Astorino, Rachel Van Cott, Jintao Guo, Drew Kortus, Wei Niu
- **Unesp_Brazil:** Paulo J. C. Freire, Danielle Biscaro Pedrolli, Nathan Vinicius Ribeiro, Bruna Fernandes Silva, Nadine Vaz Vanini, Mariana Santana da Mota, Larissa de Souza Crispim
- **UNSW_Australia:** Tyler Chapman, Tobias Gaitt, Megan Jones, Emily Watson
- **UPF_CRG_Barcelona:** Guillem Lopez-Grado, Laura Sans

- **Uppsala:** Matilda Brink, Varshni Rajagopal, Elin Ramstrom
- **US_AFRL_CarrollHS:** Anna Bete, Yazmin Camacho, Jonah Carter, Christina Davis, Jason Dong, Amy Ehrenworth, Michael Goodson, Chris Guptil, Max Herrmann, Chia Hung, Hayley Jesse, Rachel Krabacher, Dallas McDonald, Peter Menart, Travis O'Leary, Laura Polanka, Andrea Poole, Vanessa Varaljay
- **USMA-West_Point:** Alana Appel, John Cave, Liz Huuki, Matt McDonough, Channah Mills, Alex Mitropoulos, James Pruneski, Ken Wickiser
- **USP-Brazil:** Felipe Xavier Buson, Vinicius Flores, Guilherme Meira Lima, Caio Gomes Tavares Rosa
- **UST_Beijing:** Guanke Bao, Haitao Dong, Zhi Luo, Jiarong Peng
- **USTC:** Yongyan An, Cheng Cheng, Zhenyu Jiang, Linzhen Kong, Chenfei Luo, Liudong Luo, Yingying Shi, Erting Tang, Ping Wang, Yuyang Wang, Guiyang Xu, Wenfei Yu, Bonan Zhang, Qian Zhang
- **UT-Knoxville:** David Garcia, Nannan Jiang, Brandon Kristy, Ralph Laurel, Karl Leitner, Frank Loeffler, Steven Ripp, Morgan Street
- **Utrecht:** Khadija Amheine, Felix Bindt, Meine Boer, Mike Boxem, Jolijn Govers, Seino Jongkees, Lorenzo Pattiradjawane, Pim Swart, Helen Tsang, Floor de Graaf, Marjolijn ten Dam, Franca van Heijningen
- **Valencia_UPV:** Yadira Boada, Alejandro Vignoni
- **Vilnius-Lithuania:** Valentas Brasas, Aukse Gaizauskaite, Gabrielius Jakutis, Simas Jasiunas, Ieva Juskaite, Justas Ritmejeris, Dovydas Vaitkus, Tomas Venclovas, Kornelija Vitkute, Hanna Yeliseyeva, Kristina Zukauskaite, Justina Zvirblyte
- **Vilnius-Lithuania-OG:** Laurynas Karpus, Ignas Mazelis, Irmantas Rokaitis
- **Virginia:** Ngozi D. Akingbesote, Dylan Culfogienis, William Huang, Kevin Park
- **Warwick:** Janvi Ahuja, Christophe Corre, Gurpreet Dhaliwal, Rhys Evans, Kurt Hill, Olivor Holman, Alfonso Jaramillo, Alizah Khalid, Jack Lawrence, Laura Mansfield, James O'Brien, June Ong, Satya Prakash, Jonny Whiteside
- **Washington:** Karl Anderson, Emily Chun, Grace Kim, Aerilynn Nguyen, Chemay Shola, Dorsa Toghani, Angel Wong, Joanne Wong, Jay Yung
- **WashU_StLouis:** Elizabeth Johnson, Divangana Lahad, Kyle Nicholson, Havisha Pedamallu, Cam Phelan
- **Waterloo:** Clara Fikry, Leah Fulton, Nicole Lassel, Dylan Perera, Marina Robin, Nicolette Shaw
- **Westminster_UK:** Kyle Bowman, Sarah Coleman, Kristian Emilov, Camila Gaspar, Jenaagan Jenakendran, Sara Mubeen, Marko Obrvan, Caroline Smith
- **WHU-China:** Tang Bo, Du Liaoqi, Chang Tianyi, Xing Yuan, Qing Yue
- **William_and_Mary:** Eric Bradley, Stephanie Do, Xiangyi Fang, Ethan Jones, Jessica Laury, Wukun Liu, Adam Oliver, Lillian Parr, Mainak Patel, Margaret Saha, Chengwu Shen, Tinh Son, Julia Urban, Yashna Verma, Hanmi Zhou
- **Worldshaper-Wuhan:** Shan Dong, Zhengguo Hao, Yi Kuang, Ting Liu, Rui Zhou
- **WPI_Worcester:** Beck Arruda, Natalie Farny, Mei Hao, Camille Pearce, Alex Rebello, Arth Sharma, Kylie Sumner, Bailey Sweet

- **XJTLU-CHINA:** Junliang Lin
- **XJTU-China:** Du Mengtao, Fan Peiyao, Fang Xinlei
- **XMU-China:** Niangui Cai, Junhong Chen, Yousi Fu, Yunyun Hu, Ye Qiang, Qiupeng Wang, Ruofan Yang, Chen Yucheng, Jiyang Zheng
- **Yale:** Kevin Chang, Cecily Gao, Farren Isaacs, Kevin Li, Ricardo Moscoso, Jaymin Patel, Lauren Telesz, Alice Tirad
- **ZJU-China:** Qinhao Cao, Xinhua Feng, Yinjing Lu, Xianyin Zhang, Xuanhao Zhou
- **ZJUT-China:** Dongchang Sun, Zhe Yuan, Jiajie Zhou
