# Supplementary material for: Comparative analysis of three studies measuring fluorescence from engineered bacterial genetic constructs
Source: PLoS One. 2021 Jun 7;16(6):e0252263. doi: 10.1371/journal.pone.0252263 (PMC8183995; doi:10.1371/journal.pone.0252263)
Supplement: S3 File — Protocols provided to teams for use in data collection for each year and instrument. (PDF) [file pone.0252263.s003.pdf]

S2 File: Protocols

Jacob Beal, Geoff S. Baldwin, Natalie G. Farny, Markus Gershater, Traci Haddock-Angelli, Russell Buckley-Taylor, Ari Dwijayanti, Daisuke Kiga, Meagan Lizarazo, John Marken, Kim de Mora, Randy Rettberg, Vishal Sanchania, Vinoo Selvarajah, Abigail Sison, Marko Storch, Christopher T. Workman, and the iGEM Interlab Study Contributors

Contents

1 Plate Reader Protocol - 2016 **2**

2 Plate Reader Protocol - 2016 **11**

3 Plate Reader Protocol - 2017 **19**

4 Flow Cytometry Protocol - 2016 **33**

5 Flow Cytometry Protocol - 2018 **39**

Before You Begin

Read through this entire protocol sheet carefully before you start your experiment and prepare any materials you may need.

Calibration Protocols

1. OD_{600} Reference point

You will use LUDOX-S30 as a single point reference to obtain a ratiometric conversion factor to transform your absorbance data into a standard OD_{600} measurement. This has two key objectives. With standard 1 cm pathlength spectrophotometers, the reading is still instrument dependent (see above). With plate readers the path length is less than 1 cm and is volume dependent. In this instance the ratiometric conversion can both transform Abs_{600} measurements (i.e. the basic output of the instrument and not standardised optical density with 1 cm pathlength) into OD_{600} measurements, whilst simultaneously accounting for instrument differences.

[**IMPORTANT NOTE**: many plate readers have an automatic path length correction, this is based on volume adjustment using a ratio of absorbance measurements at 900 and 950 nm. Because scattering increases with longer wavelengths, this adjustment is confounded by scattering solutions, such as dense cells. **YOU MUST THEREFORE TURN OFF PATHLENGTH CORRECTION**.]

To measure your standard LUDOX Abs_{600} you must use the same cuvettes, plates and volumes (**suggestion**: use 100 μl for plate reader measurement and 1 mL for spectrophotometer measurement) that you will use in your cell based assays. The LUDOX solution is only weakly scattering and so will give a low absorbance value.

If using plates prepare a column of 4 wells with 100 μl 100% LUDOX and 4 wells containing 100 μl H_2O. Repeat the measurement in all relevant modes used in your experiments (e.g. settings for orbital averaging).

If using a cuvette, you will only have enough material for a single measurement, but repeat the reading multiple times. Use the same cuvette to measure the reference with H_2O (this value will be subtracted by the instrument to give the OD_{600} reading).

Materials:

1ml LUDOX (provided in kit)
H_20 (provided by team)
96 well plate or cuvettes (provided by team)

Method

- ☐ Add 100 µl LUDOX into wells A1, B1, C1, D1 (or 1 mL LUDOX into cuvette)
- ☐ Add 100 µl of H_2O into wells A2, B2, C2, D2 (or 1 mL H_2O into cuvette)
- ☐ Measure absorbance 600 nm of all samples in all standard measurement modes in instrument
- ☐ Record the data in the table below or in your notebook
- ☐ Import data into Excel (**OD600 reference point tab**) Sheet_1 provided

	LUDOX 100%	H_2O
replicate 1		
replicate 2		
replicate 3		
replicate 4		

Example data:

	A	B	C	D	Average	Corrected Abs_{600}	Reference OD_{600}	Correction Factor
**Spectrophotometer**	0.016	0.014	0.014	0.015	0.01475	0.01475	0.01475	1
**Plate reader H_2O**	0.033	0.033	0.034	0.033	0.03375	0		
**Plate reader LUDOX**	0.041	0.041	0.041	0.041	0.041	0.00775	0.01475	1.903

Table shows the data for OD_{600} measured in the reference spectrophotometer. For the plate reader data we show the measured Abs_{600} for the H_2O and LUDOX. The corrected Abs_{600} is calculated by subtracting the H_2O reading. The reference OD_{600} is defined as that measured by the reference spectrophotometer (you should use this value too). The correction factor to convert measured Abs_{600} to OD_{600} is thus the Reference OD_{600} divided by Abs_{600}. All cell density readings using this instrument with the same settings and volume can be converted to OD_{600} by multiplying by (in this instance) 1.903.

2. Protocol FITC fluorescence standard curve

You will prepare a dilution series of FITC in 4 replicates and measure the fluorescence in a 96 well plate in your plate reader or individually in cuvettes in a fluorimeter. By measuring these in all standard modes in your plate reader or fluorimeter, you will generate a standard curve of fluorescence for FITC concentration. You will be able to use this to correct your cell based readings to an equivalent fluorescein concentration. You will then be able to convert this into a concentration of GFP.

Before beginning this protocol ensure that you are familiar with the GFP settings and measurement modes of your instrument.

Materials:

194.7 μg FITC (provided in kit)
10ml 1xPBS (phosphate buffered saline; provided by team)
96 well plate or cuvettes (provided by team)

Method

Prepare the FITC stock solution:

- ☐ Spin down FITC stock tube to make sure pellet is at the bottom of tube.
- ☐ Prepare 2x FITC stock solution (500 μM) by resuspending FITC in 1 mL of 1xPBS
- ☐ Incubate the solution at 42°C for 4 hours
- ☐ Dilute the 2x FITC stock solution in half with 1xPBS to make a 1x FITC solution and resulting concentration of FITC stock solution 250 μM.

[**Note**: it is important that the FITC is properly dissolved. To check this after the incubation period pipetted up and down – if any particulates are visible in the pipette tip continue to incubate overnight.]

Prepare the serial dilutions of FITC:

Accurate pipetting is essential. Serial dilutions will be performed across columns 1-11. COLUMN 12 MUST CONTAIN PBS BUFFER ONLY. Initially you will setup the plate with the FITC stock in column 1 and an equal volume of 1xPBS in columns 2 to 12. You will perform a serial dilution by consecutively transferring 100 μl from column to column with good mixing.

Overview samples in 96 well plate or cuvettes

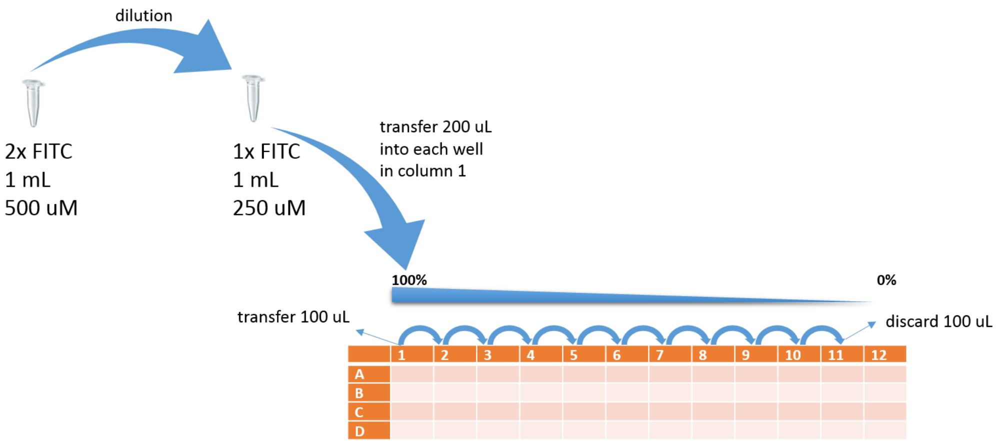

- ☐ **Add 100 µl of PBS** into wells A2, B2, C2, D2....A12, B12, C12, D12
- ☐ **Add 200 µl of FITC 1x stock** solution into A1, B1, C1, D1
- ☐ Transfer 100 µl of FITC stock solution from A1 into A2.
- ☐ Mix A2 by pipetting up and down 3x and transfer 100 µl into A3…
- ☐ Mix A3 by pipetting up and down 3x and transfer 100 µl into A4...
- ☐ Mix A4 by pipetting up and down 3x and transfer 100 µl into A5...
- ☐ Mix A5 by pipetting up and down 3x and transfer 100 µl into A6...
- ☐ Mix A6 by pipetting up and down 3x and transfer 100 µl into A7...
- ☐ Mix A7 by pipetting up and down 3x and transfer 100 µl into A8...
- ☐ Mix A8 by pipetting up and down 3x and transfer 100 µl into A9...
- ☐ Mix A9 by pipetting up and down 3x and transfer 100 µl into A10...
- ☐ Mix A10 by pipetting up and down 3x and transfer 100 µl into A11...
- ☐ Mix A11 by pipetting up and down 3x and transfer 100 µl into **liquid waste**

TAKE CARE NOT TO CONTINUE SERIAL DILUTION INTO COLUMN 12.

- ☐ Repeat dilution series for rows B, C, D
- ☐ Measure fluorescence of all samples in all standard measurement modes in instrument
- ☐ Record the data in your notebook
- ☐ Import data into Excel (**FITC standard curve tab**) Sheet_1 provided

For cuvette usage:

Use 2.0mL tubes for cuvette dilutions, and then transfer 1.0mL into your cuvettes.

- ☐ **Add 1 mL of PBS** into tubes 2-11.
- ☐ **Add 2.0 mL of FITC 1x stock** solution tube 1
- ☐ Transfer 1.0 ml of FITC stock solution tube 1 into tube 2
- ☐ Mix tube 2 by pipetting up and down 3x and transfer 1 mL into tube 3…

- ☐ Mix tube 3 by pipetting up and down 3x and transfer 1 mL into tube 4…
- ☐ Mix tube 4 by pipetting up and down 3x and transfer 1 mL into tube 5…
- ☐ Mix tube 5 by pipetting up and down 3x and transfer 1 mL into tube 6…
- ☐ Mix tube 6 by pipetting up and down 3x and transfer 1 mL into tube 7…
- ☐ Mix tube 7 by pipetting up and down 3x and transfer 1 mL into tube 8…
- ☐ Mix tube 8 by pipetting up and down 3x and transfer 1 mL into tube 9…
- ☐ Mix tube 9 by pipetting up and down 3x and transfer 1 mL into tube 10…
- ☐ Mix tube 10 by pipetting up and down 3x and transfer 1 mL into tube 11…
- ☐ Mix tube 11 by pipetting up and down 3x and and transfer 1 mL into **liquid waste**

Measurement Notes

You must now measure the plate (or cuvettes) in your plate reader (or fluorimeter). The machine must be setup with the standard GFP settings (filters or excitation and emission wavelengths) that you will use when measuring your cells (if you change them you will not be able to use this standard curve). It is therefore a good idea to repeat the measurement a number of times with different settings. You will then have a series of standard curves to choose from. Most important it is necessary to use a number of settings that affect the sensitivity (principally gain and/or slit width). Be sure to also consider other options (orbital averaging, top/bottom optics). As before, TURN OFF path length correction if available.

Make sure to record all information about your instrument (wavelengths, etc.) as these will be required in the Plate Reader Form.

Cell measurement protocol

Prior to performing the measurement on the cells you should perform the **calibration measurements**. This will ensure that you understand the measurement process and that you can take the cell measurements under the same conditions.

Materials:

Competent cells (ideally *Escherichia coli* strain DH5α or TOP10)
Terrific broth (at half strength: 0.5x TB) or can use LB (Luria Bertani) media as an alternative
Chloramphenicol (stock concentration 25 mg/mL dissolved in EtOH)
50 ml Falcon tube (or equivalent) or 250 ml shake flask for cell growth
Incubator at 37°C
1.5 ml eppendorf tubes for sample storage
Ice bucket with ice
Pipettes

Devices (from InterLab Measurement Kit):

- Positive control
- Negative control
- Device 1: J23101+I13504
- Device 2: J23106+I13504
- Device 3: J23117+I13504

Workflow

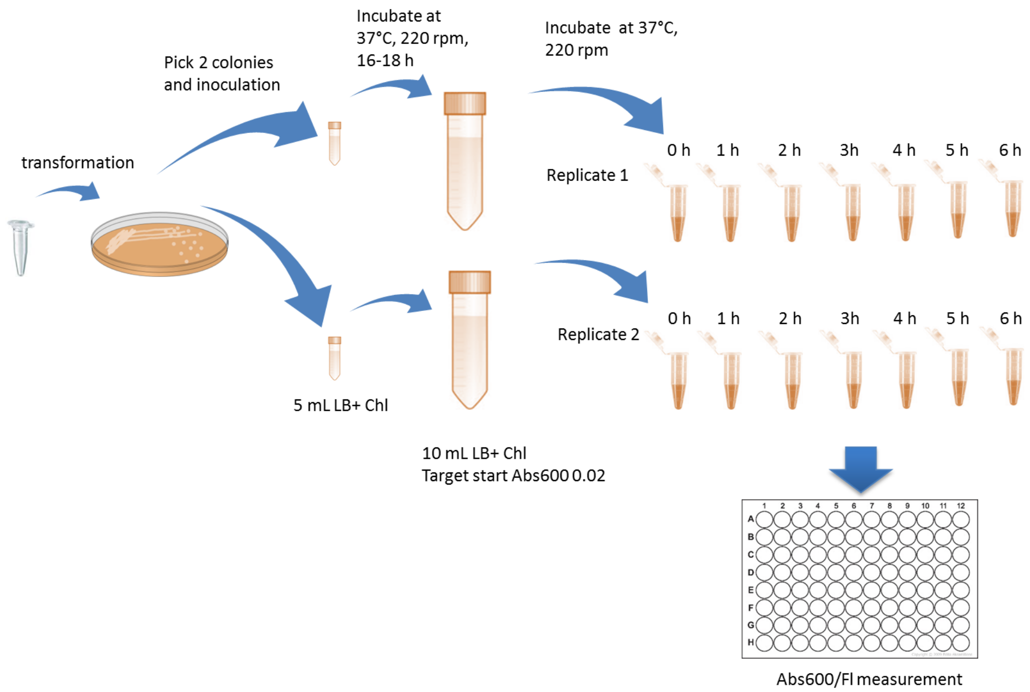

Method

Day 1: transform *Escherichia coli* DH5α or TOP10 with these following plasmids:

- Positive control
- Negative control
- Device 1: J23101+I13504
- Device 2: J23106+I13504
- Device 3: J23117+I13504

Day 2: Pick 2 colonies from each of plate and inoculate it on 5-10 mL LB medium + Chloramphenicol. Grow the cells overnight (16-18 hours) at 37°C and 220 rpm.

Day 3: Cell growth, sampling, and assay

Note the differences, depending on whether you are making your readings in a plate reader or 1 ml cuvettes:

- ☐ Set your instrument to read OD600 (as OD calibration setting)
- ☐ Measure OD600 of the overnight cultures
- ☐ Record data in your notebook
- ☐ Import data into Excel (**normalisation tab**) Sheet_1 provided
- ☐ Dilute the cultures to a target OD_{600} of 0.02 (see the volume of preloading culture and media in Excel (**normalisation tab**) Sheet_1) in 10 ml 0.5x TB medium +

Chloramphenicol in 50 mL falcon tube (if using cuvettes, you can use 100 ml in a 500 ml shake flask).

- ☐ Incubate the cultures at 37°C and 220 rpm.
- ☐ Take 100 µL (1% of total volume) samples of the cultures at 0, 1, 2, 3, 4, 5, and 6 hours of incubation (if using cuvettes, remove 1 ml from 100 ml culture).
- ☐ Place samples on ice.
- ☐ At the end of sampling point you need to measure your samples (OD and Fl measurement), see the below for details.
- ☐ Record data in your notebook
- ☐ Import data into Excel (**cell measurement tab**) Sheet_1 provided

Measurement

It is important that you use the same instrument settings that you used when measuring the FITC standard curve. <u>This includes using the sample volume (100 ul) or 1 mL sample for measurement using spectrophotometer.</u>

Samples should be laid out according to Fig. 2. Pipette 100 µl of each sample into each well. Set the instrument settings as those that gave the best results in your calibration curves (no measurements off scale). If necessary you can test more than one of the previously calibrated settings to get the best data (no measurements off scale).

Hint:
No measurement off scale means the data you get does not out of range of your calibration curve.

Layout for Abs600 and Fluorescence measurement

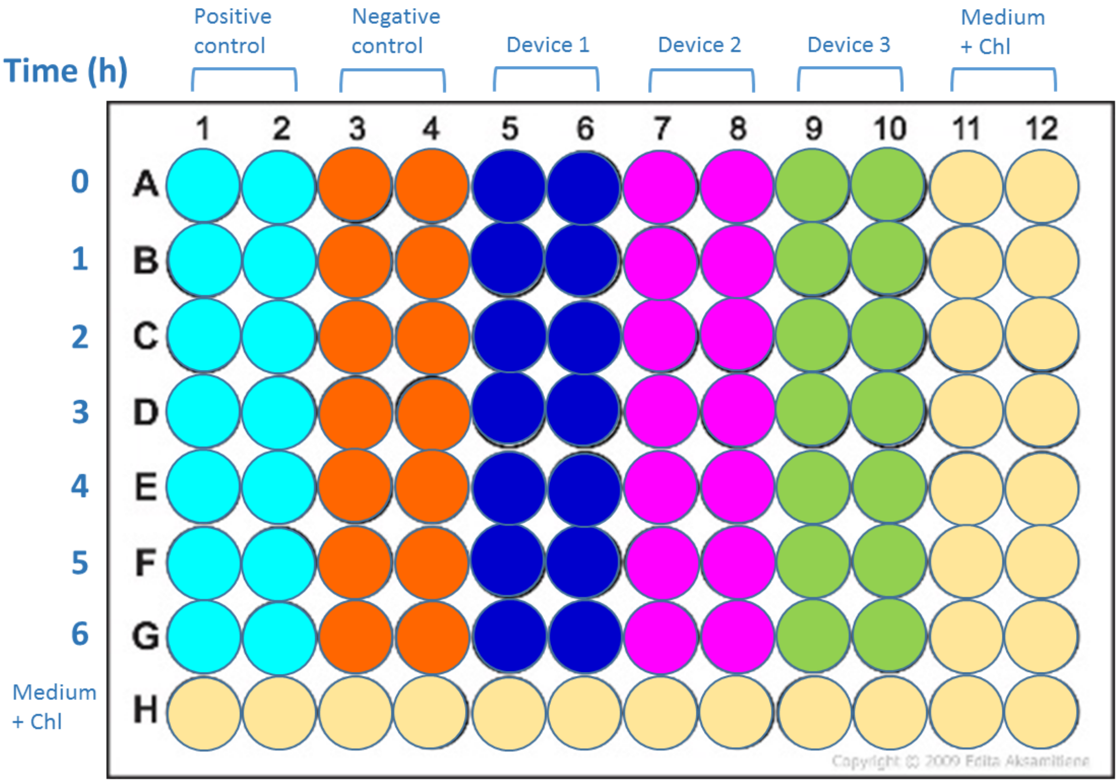
Time (h)
Positive control
Negative control
Device 1
Device 2
Device 3
Medium + Chl
1
2
3
4
5
6
7
8
9
10
11
12
0
A
1
B
2
C
3
D
4
E
5
F
6
G
Medium + Chl
H
Copyright © 2009 Edita Aksamitiene

Before You Begin

Read through this entire protocol sheet carefully before you start your experiment and prepare any materials you may need. This year, in order to improve reproducibility, we are requiring all participating teams to use plate readers to take their measurements. If you do not have access to a plate reader, you may collaborate with another team. If the plate reader requirement is a significant barrier for your team, you can still participate in the InterLab study. Contact the iGEM Measurement Committee at measurement at igem dot org to discuss your situation.

Calibration Protocols

1. OD_{600} Reference point

You will use LUDOX-S40 as a single point reference to obtain a ratiometric conversion factor to transform your absorbance data into a standard OD_{600} measurement. This has two key objectives. With standard 1 cm pathlength spectrophotometers, the reading is still instrument dependent (see above). With plate readers the path length is less than 1 cm and is volume dependent. In this instance the ratiometric conversion can both transform Abs_{600} measurements (i.e. the basic output of the instrument and not standardised optical density with 1 cm pathlength) into OD_{600} measurements, whilst simultaneously accounting for instrument differences.

[**IMPORTANT NOTE**: many plate readers have an automatic path length correction, this is based on volume adjustment using a ratio of absorbance measurements at 900 and 950 nm. Because scattering increases with longer wavelengths, this adjustment is confounded by scattering solutions, such as dense cells. **YOU MUST THEREFORE TURN OFF PATHLENGTH CORRECTION**.]

To measure your standard LUDOX Abs_{600} you must use the same cuvettes, plates and volumes (**suggestion**: use 100 µl for plate reader measurement and 1 mL for spectrophotometer measurement) that you will use in your cell based assays. The LUDOX solution is only weakly scattering and so will give a low absorbance value.

Prepare a column of 4 wells with 100 µl 100% LUDOX and 4 wells containing 100 µl H_2O. Repeat the measurement in all relevant modes used in your experiments (e.g. settings for orbital averaging).

Materials:

1ml LUDOX (provided in kit)
H_20 (provided by team)
96 well plate, black with flat, transparent/clear bottom preferred (provided by team)

Method

- ☐ Add 100 µl LUDOX into wells A1, B1, C1, D1 (or 1 mL LUDOX into cuvette)
- ☐ Add 100 µl of H_2O into wells A2, B2, C2, D2 (or 1 mL H_2O into cuvette)
- ☐ Measure absorbance 600 nm of all samples in all standard measurement modes in instrument
- ☐ Record the data in the table below or in your notebook
- ☐ Import data into Excel (**OD600 reference point tab**) Sheet_1 provided

	LUDOX 100%	H_2O
replicate 1		
replicate 2		
replicate 3		
replicate 4		

Example Data:

	reference spectrophotometer		microplate reader	
	H20	LDX-HS40 100%	H20	LDX-HS40 100%
replicate 1	0	0.043	0.036	0.112
replicate 2	0	0.043	0.034	0.078
replicate 3	0	0.042	0.033	0.095
replicate 4	0	0.042	0.035	0.101
average	0	0.0425	0.0345	0.0965
corrected Abs600		0.0425		0.062
reference OD600		0.0425		0.0425
correction factor		1		0.685483871

Table shows the data for OD_{600} measured by a spectrophotometer and a plate reader for the H_2O and LUDOX. The corrected Abs_{600} is calculated by subtracting the H_2O reading. The reference OD_{600} is defined as that measured by the reference spectrophotometer (you should use this value too). The correction factor to convert measured Abs_{600} to OD_{600} is thus the Reference OD_{600} divided by Abs_{600}. ***All cell density readings using this instrument with the same settings and volume can be converted to*** OD_{600} ***by multiplying by (in this instance) 0.685.***

2. Protocol fluorescein fluorescence standard curve

You will prepare a dilution series of fluorescein in 4 replicates and measure the fluorescence in a 96 well plate in your plate reader. By measuring these in all standard modes in your plate reader, you will generate a standard curve of fluorescence for fluorescein concentration. You will be able to use this to correct your cell based readings to an equivalent fluorescein concentration. You will then be able to convert this into a concentration of GFP.

Before beginning this protocol ensure that you are familiar with the GFP settings and measurement modes of your instrument.

Materials:

fluorescein (provided in kit)
10ml 1xPBS (phosphate buffered saline; provided by team)
96 well plate, black with flat, transparent/clear bottom preferred (provided by team)

Method

Prepare the fluorescein stock solution:

- ☐ Spin down fluorescein stock tube to make sure pellet is at the bottom of tube.
- ☐ Prepare 2x fluorescein stock solution (100 µM) by resuspending fluorescein in 1 mL of 1xPBS. [**Note**: it is important that the fluorescein is properly dissolved. To check this, after the resuspension you should pipette up and down and examine the solution in the pipette tip – if any particulates are visible in the pipette tip continue to mix the solution until they disappear.]
- ☐ Dilute the 2x fluorescein stock solution with 1xPBS to make a 1x fluorescein solution and resulting concentration of fluorescein stock solution 50 µM (500µL of 2x fluorescein in 500 µL 1x PBS will make 1 mL of 50 µM (1x) fluorescein solution.)

Prepare the serial dilutions of fluorescein:

Accurate pipetting is essential. Serial dilutions will be performed across columns 1-11. COLUMN 12 MUST CONTAIN PBS BUFFER ONLY. Initially you will setup the plate with the fluorescein stock in column 1 and an equal volume of 1xPBS in columns 2 to 12. You will perform a serial dilution by consecutively transferring 100 µl from column to column with good mixing.

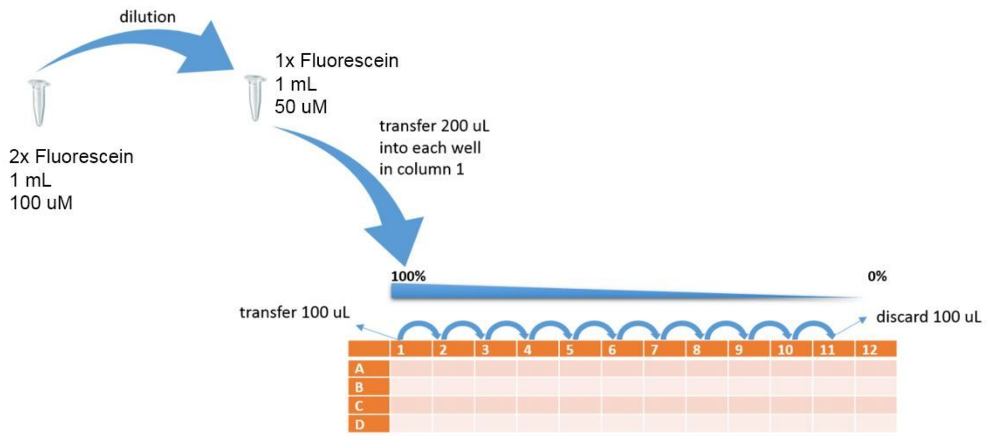

Overview of 96-well plate setup

- ☐ **Add 100 µl of PBS** into wells A2, B2, C2, D2....A12, B12, C12, D12
- ☐ **Add 200 µl of fluorescein 1x stock** solution into A1, B1, C1, D1
- ☐ Transfer 100 µl of fluorescein stock solution from A1 into A2.
- ☐ Mix A2 by pipetting up and down 3x and transfer 100 µl into A3…
- ☐ Mix A3 by pipetting up and down 3x and transfer 100 µl into A4...
- ☐ Mix A4 by pipetting up and down 3x and transfer 100 µl into A5...
- ☐ Mix A5 by pipetting up and down 3x and transfer 100 µl into A6...
- ☐ Mix A6 by pipetting up and down 3x and transfer 100 µl into A7...
- ☐ Mix A7 by pipetting up and down 3x and transfer 100 µl into A8...
- ☐ Mix A8 by pipetting up and down 3x and transfer 100 µl into A9...
- ☐ Mix A9 by pipetting up and down 3x and transfer 100 µl into A10...
- ☐ Mix A10 by pipetting up and down 3x and transfer 100 µl into A11...
- ☐ Mix A11 by pipetting up and down 3x and transfer 100 µl into **liquid waste**

TAKE CARE NOT TO CONTINUE SERIAL DILUTION INTO COLUMN 12.

- ☐ Repeat dilution series for rows B, C, D
- ☐ Measure fluorescence of all samples in all standard measurement modes in instrument
- ☐ Record the data in your notebook
- ☐ Import data into Excel (fluorescein **standard curve tab**) Sheet_1 provided

Measurement Notes

You must now measure the plate in your plate reader. The machine must be setup with the standard GFP settings (filters or excitation and emission wavelengths) that you will use when measuring your

cells (if you change them you will not be able to use this standard curve). It is therefore a good idea to repeat the measurement a number of times with different settings. You will then have a series of standard curves to choose from. Most important it is necessary to use a number of settings that affect the sensitivity (principally gain and/or slit width). Be sure to also consider other options (orbital averaging, top/bottom optics). As before, TURN OFF path length correction if available.

Make sure to record all information about your instrument (wavelengths, etc.) as these will be required in the Plate Reader Form.

Recommended filters:

Excitation 485nm

Emission 530/30 (or as close to this as possible)

Cell measurement protocol

Prior to performing the measurement on the cells you should perform the **calibration measurements**. This will ensure that you understand the measurement process and that you can take the cell measurements under the same conditions. This year, for the sake of consistency and reproducibility, we are requiring all teams to use *E. coli* K-12 DH5-alpha. If you do not have access to this strain, you can request streaks of the transformed devices from another team near you, and this can count as a collaboration as long as its appropriately documented on both team's wikis. If you are absolutely unable to obtain the DH5-alpha strain, you may still participate in the InterLab study by contacting the Measurement Committee (measurement at igem dot org) to discuss your situation.

Materials:

Competent cells (*Escherichia coli* strain DH5 α)
LB (Luria Bertani) media
Chloramphenicol (stock concentration 25 mg/mL dissolved in EtOH - working stock 25 ug/mL)
50 ml Falcon tube (or equivalent, preferably amber or covered in foil to block light)
Incubator at 37°C
1.5 ml eppendorf tubes for sample storage
Ice bucket with ice
Pipettes
96 well plate, black with flat, transparent/clear bottom preferred (provided by team)

Devices (from InterLab Measurement Kit):

- Positive control
- Negative control
- Test Device 1: J23101+I13504
- Test Device 2: J23106+I13504
- Test Device 3: J23117+I13504
- Test Device 4: J23101.BCD2.E0040.B0015
- Test Device 5: J23106.BCD2.E0040.B0015
- Test Device 6: J23117.BCD2.E0040.B0015

Workflow

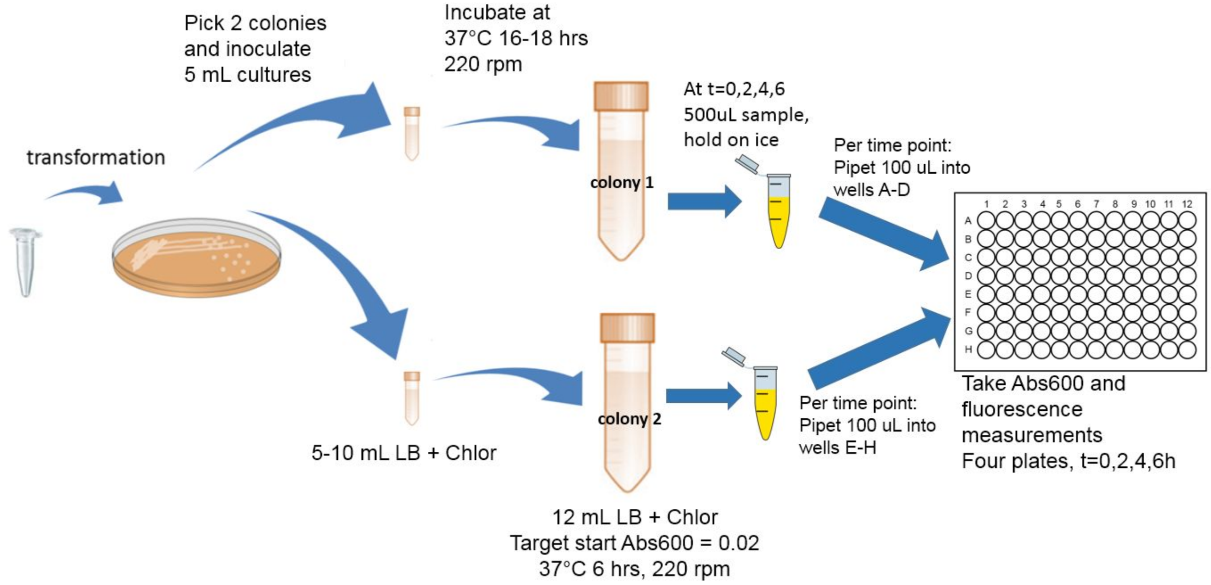

Method

Day 1: transform *Escherichia coli* DH5 α with these following plasmids:

- Positive control
- Negative control
- Test Device 1: J23101+I13504
- Test Device 2: J23106+I13504
- Test Device 3: J23117+I13504
- Test Device 4: J23101.BCD2.E0040.B0015
- Test Device 5: J23106.BCD2.E0040.B0015
- Test Device 6: J23117.BCD2.E0040.B0015

Day 2: Pick 2 colonies from each of plate and inoculate it on 5-10 mL LB medium + Chloramphenicol. Grow the cells overnight (16-18 hours) at 37°C and 220 rpm.

Day 3: Cell growth, sampling, and assay

- ☐ Set your instrument to read OD600 (as OD calibration setting)
- ☐ Measure OD600 of the overnight cultures
- ☐ Record data in your notebook
- ☐ Import data into Excel (**Dilution Calculation**) Sheet_1 provided
- ☐ Dilute the cultures to a target OD_{600} of 0.02 (see the volume of preloading culture and media in Excel (**Dilution Calculation**) Sheet_1) in **12 ml** LB medium + Chloramphenicol in 50 mL falcon tube (amber, or covered with foil to block light).
- ☐ Incubate the cultures at 37°C and 220 rpm.
- ☐ Take 500 µL samples of the cultures at 0, 2, 4, and 6 hours of incubation. (At each time point, you will take a sample from each of the 8 devices, two colonies per device, for a total of 16 samples per time point)
- ☐ Place samples on ice.
- ☐ At the end of sampling point you need to measure your samples (OD and Fl measurement), see the below for details.
- ☐ Record data in your notebook
- ☐ Import data into Excel (**cell measurement tab**) Sheet_1 provided

Measurement

It is important that you use the same instrument settings that you used when measuring the fluorescein standard curve. This includes using the sample volume (100 ul) you used for the fluorescein measurement.

Samples should be laid out according to Fig. 2. Pipette 100 µl of each sample into each well. Replicate samples of colony #1 should be pipetted into wells in rows A, B, C and D. Replicate

samples of colony #2 should be pipetted into wells in rows E, F, G and H. Set the instrument settings as those that gave the best results in your calibration curves (no measurements off scale). If necessary you can test more than one of the previously calibrated settings to get the best data (no measurements off scale).

Hint:
No measurement off scale means the data you get does not out of range of your calibration curve.

Layout for Abs600 and Fluorescence measurement

At the end of the experiment, you should have four plates to read. Each plate should be set up as shown below. You will have one plate for each time point: 0, 2, 4, and 6 hours.

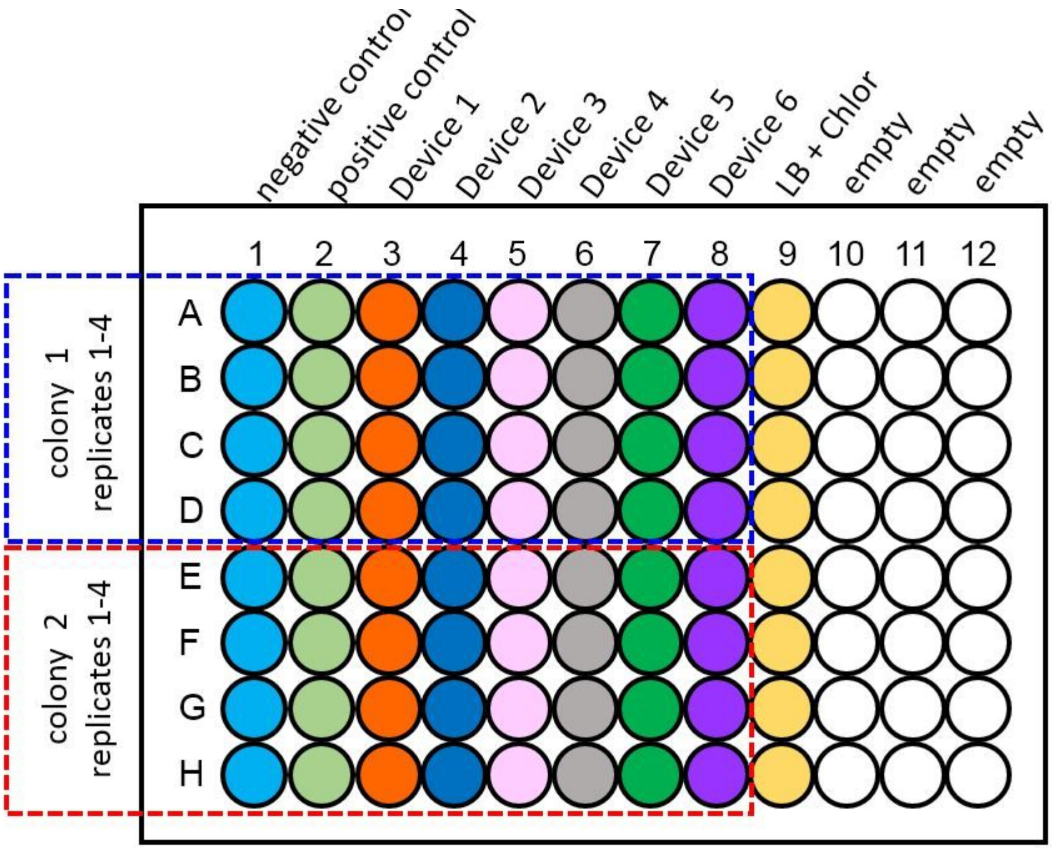

iGEM 2018 InterLab Study Protocol

Before You Begin

Read through this entire protocol sheet carefully before you start your experiment and prepare any materials you may need. In order to improve reproducibility, **we are requiring all participating teams to use plate readers to take measurements of fluorescence and absorbance**. If you do not have access to a plate reader with those capabilities, you may collaborate with another team. If the plate reader requirement is a significant barrier for your team, you can still participate in the InterLab study. Contact the iGEM Measurement Committee at measurement at igem dot org to discuss your situation.

Before beginning your experiments, it will be helpful to gather the following information about your plate reader, as you will be asked to provide this information when submitting your data to iGEM HQ:

- ❏ Instrument brand and model ____
- ❏ Can your instrument measure both absorbance and fluorescence? ________________
- ❏ Does your instrument have pathlength correction, and if yes can it be disabled? ____
- ❏ Does your instrument have variable temperature settings, and if yes can this be set to room temperature? ____
- ❏ What filters does your instrument have for measuring GFP? You will need information about the bandpass width (530 nm / 30 nm bandpass, 25-30nm width is recommended), excitation (485 nm is recommended) and emission (520-530 nm is recommended) of this filter. ____
- ❏ Does your instrument use top or bottom optics (i.e. does your plate reader read samples from the top of the plate or the bottom)? _____________________________

You will need all of the following supplies and reagents to complete this entire protocol. Please take a moment to check that you have all of these supplies and reagents before you begin:

- ❏ Measurement Kit (provided with the iGEM distribution shipment) containing:
    - ❏ 1ml LUDOX CL-X
    - ❏ 150 µL Silica Bead (microsphere suspension)
    - ❏ Fluorescein (powder, in amber tube)

- ❏ iGEM Parts Distribution Kit Plates (you will obtain the test devices from the parts kit plates)
- ❏ 1x PBS (phosphate buffered saline, pH 7.4 - 7.6)
- ❏ ddH_2O (ultrapure filtered or double distilled water)
- ❏ Competent cells (*Escherichia coli* strain DH5 α)
- ❏ LB (Luria Bertani) media
- ❏ Chloramphenicol (stock concentration 25 mg/mL dissolved in EtOH)
- ❏ 50 ml Falcon tube (or equivalent, preferably amber or covered in foil to block light)
- ❏ Incubator at 37°C
- ❏ 1.5 ml eppendorf tubes
- ❏ Ice bucket with ice
- ❏ Micropipettes (capable of pipetting a range of volumes between 1 µL and 1000 µL)
- ❏ Micropipette tips
- ❏ 96 well plates, black with clear flat bottom preferred, at least 3-4 plates (provided by team)

Calibration Protocols

CALIBRATION PROTOCOLS SHOULD BE COMPLETED BEFORE CELL MEASUREMENTS ARE TAKEN!

You will make three sets of unit calibration measurements: an OD_{600} reference point, a particle standard curve, and a fluorescein standard curve. Before beginning these protocols, please ensure that you are familiar with the measurement modes and settings of your instrument.

For all of these calibration measurements, you must use the same plates and volumes that you will use in your cell-based assays. You must also use the same settings (e.g., filters or excitation and emission wavelengths) that you will use in your cell-based assays. **If you do not use the same plates, volumes, and settings, the calibration will not be valid.** Make sure to record all information about your instrument (checklist on page 1 of this protocol) as these will be required later when you document your experiment. If your instrument has variable temperature settings, the instrument temperature should be set to room temperature (approximately 20-25 C) for all measurements.

Calibration 1: OD_{600} Reference point - LUDOX Protocol

You will use LUDOX CL-X (45% colloidal silica suspension) as a single point reference to obtain a conversion factor to transform your absorbance (Abs_{600}) data from your plate reader into a comparable OD_{600} measurement as would be obtained in a spectrophotometer. Such conversion is

necessary because plate reader measurements of absorbance are volume dependent; the depth of the fluid in the well defines the path length of the light passing through the sample, which can vary slightly from well to well. In a standard spectrophotometer, the path length is fixed and is defined by the width of the cuvette, which is constant. Therefore this conversion calculation can transform Abs_{600} measurements from a plate reader (i.e., absorbance at 600nm, the basic output of most instruments) into comparable OD_{600} measurements. The LUDOX solution is only weakly scattering and so will give a low absorbance value.

[**IMPORTANT NOTE**: many plate readers have an automatic path length correction feature. This adjustment compromises the accuracy of measurement in highly light scattering solutions, such as dense cultures of cells. **YOU MUST THEREFORE TURN OFF PATHLENGTH CORRECTION if it can be disabled on your instrument**.]

Materials:

1ml LUDOX CL-X (provided in kit)
ddH_20 (provided by team)
96 well plate, black with clear flat bottom preferred (provided by team)

Method

- ❏ Add 100 µl LUDOX into wells A1, B1, C1, D1
- ❏ Add 100 µl of dd H_2O into wells A2, B2, C2, D2
- ❏ Measure absorbance at 600 nm of all samples in the measurement mode you plan to use for cell measurements
- ❏ Record the data in the table below or in your notebook
- ❏ Import data into Excel sheet provided (**OD600 reference point tab**)

	LUDOX CL-X	ddH_2O
replicate 1		
replicate 2		
replicate 3		
replicate 4		

	A	B	C	D
1		LUDOX CL-X	H2O	
2	Replicate 1	0.078	0.038	
3	Replicate 2	0.077	0.038	
4	Replicate 3	0.078	0.038	
5	Replicate 4	0.078	0.038	
6	Arith. Mean	0.078	0.038	
7	Corrected Abs600	0.040		
8	Reference OD600	0.063		
9	OD600/Abs600	1.585		
10				
11				

The screen capture image above is from the OD600 Reference Point tab of the InterLab Excel sheet. The table shows the data for OD_{600} measured by a spectrophotometer (row 8, yellow box, "Reference OD600") and plate reader data for the H_2O and LUDOX similar to what you will likely collect (you will place your own data in the blue boxes). The corrected Abs_{600} is calculated by subtracting the H_2O reading. The reference OD_{600} is defined as that measured by the reference spectrophotometer (as provided to you in the Excel sheet). The correction factor to convert measured Abs_{600} to OD_{600} is thus the Reference OD_{600} divided by Abs_{600}. ***All cell density readings using this instrument with the same settings and volume can be converted to OD_{600} by multiplying by (in this example) 1.585.***

<u>Calibration 2:</u> Particle Standard Curve - Microsphere Protocol

You will prepare a dilution series of monodisperse silica microspheres and measure the Abs_{600} in your plate reader. The size and optical characteristics of these microspheres are similar to cells, and there is a known amount of particles per volume. This measurement will allow you to construct a standard curve of particle concentration which can be used to convert Abs_{600} measurements to an estimated number of cells.

Materials:

300 µL Silica beads - Microsphere suspension (provided in kit, 4.7 x 10^8 microspheres)
ddH_20 (provided by team)
96 well plate, black with clear flat bottom preferred (provided by team)

Method:

Prepare the Microsphere Stock Solution:

❑ Obtain the tube labeled "Silica Beads" from the InterLab test kit and vortex

vigorously for 30 seconds. **NOTE: Microspheres should NOT be stored at 0°C or below**, as freezing affects the properties of the microspheres. If you believe your microspheres may have been frozen, please contact the iGEM Measurement Committee for a replacement (measurement at igem dot org).

- ❑ Immediately pipet 96 µL microspheres into a 1.5 mL eppendorf tube
- ❑ Add 904 µL of ddH_2O to the microspheres
- ❑ Vortex well. This is your Microsphere Stock Solution.

Prepare the serial dilution of Microspheres:

Accurate pipetting is essential. Serial dilutions will be performed across columns 1-11. COLUMN 12 MUST CONTAIN ddH_2O ONLY. Initially you will setup the plate with the microsphere stock solution in column 1 and an equal volume of 1x ddH_2O in columns 2 to 12. You will perform a serial dilution by consecutively transferring 100 µl from column to column with good mixing.

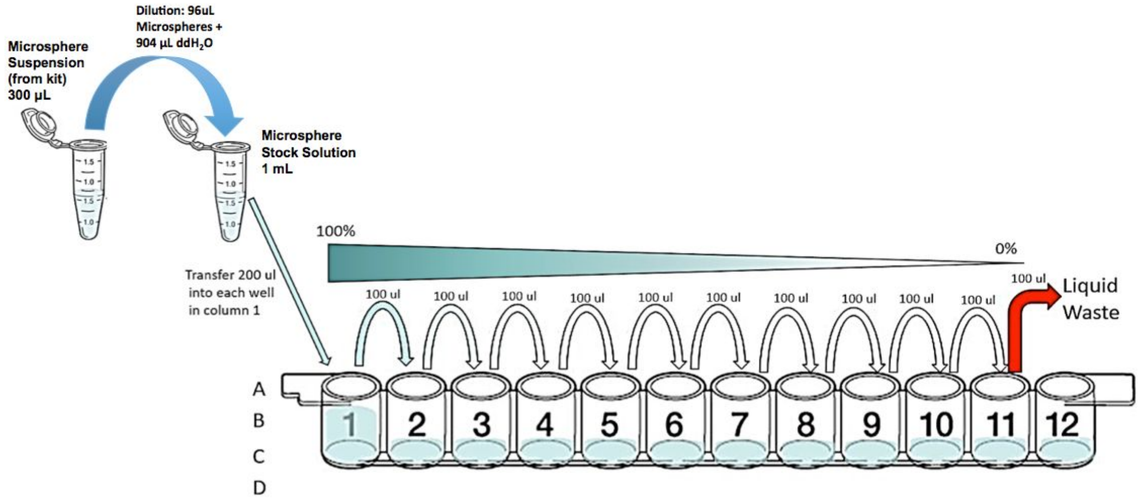

- ❑ **Add 100 µl of ddH_2O** into wells A2, B2, C2, D2....A12, B12, C12, D12
- ❑ **Vortex the tube containing the stock solution of microspheres vigorously for 10 seconds**
- ❑ **Immediately add 200 µl of microspheres stock solution into A1**
- ❑ Transfer 100 µl of microsphere stock solution from A1 into A2.
- ❑ Mix A2 by pipetting up and down 3x and transfer 100 µl into A3…
- ❑ Mix A3 by pipetting up and down 3x and transfer 100 µl into A4...
- ❑ Mix A4 by pipetting up and down 3x and transfer 100 µl into A5...
- ❑ Mix A5 by pipetting up and down 3x and transfer 100 µl into A6...
- ❑ Mix A6 by pipetting up and down 3x and transfer 100 µl into A7...
- ❑ Mix A7 by pipetting up and down 3x and transfer 100 µl into A8...

- ❑ Mix A8 by pipetting up and down 3x and transfer 100 µl into A9...
- ❑ Mix A9 by pipetting up and down 3x and transfer 100 µl into A10...
- ❑ Mix A10 by pipetting up and down 3x and transfer 100 µl into A11...
- ❑ Mix A11 by pipetting up and down 3x and transfer 100 µl into **liquid waste**

TAKE CARE NOT TO CONTINUE SERIAL DILUTION INTO COLUMN 12.

- ❑ Repeat dilution series for rows B, C, D
- ❑ **IMPORTANT!** Re-Mix (Pipette up and down) each row of your plate ***immediately before*** putting in the plate reader! (This is important because the beads begin to settle to the bottom of the wells within about 10 minutes, which will affect the measurements.) <u>Take care to mix gently and avoid creating bubbles on the surface of the liquid.</u>
- ❑ Measure Abs_{600} of all samples in instrument
- ❑ Record the data in your notebook
- ❑ Import data into Excel sheet provided (**particle standard curve tab**)

<u>Calibration 3:</u> Fluorescence standard curve - Fluorescein Protocol

Plate readers report fluorescence values in arbitrary units that vary widely from instrument to instrument. Therefore absolute fluorescence values cannot be directly compared from one instrument to another. In order to compare fluorescence output of test devices between teams, it is necessary for each team to create a standard fluorescence curve. Although distribution of a known concentration of GFP protein would be an ideal way to standardize the amount of GFP fluorescence in our *E. coli* cells, the stability of the protein and the high cost of its purification are problematic. We therefore use the small molecule fluorescein, which has similar excitation and emission properties to GFP, but is cost-effective and easy to prepare. (The version of GFP used in the devices, GFP mut3b, has an excitation maximum at 501 nm and an emission maximum at 511 nm; fluorescein has an excitation maximum at 494 nm and an emission maximum at 525nm).

You will prepare a dilution series of fluorescein in four replicates and measure the fluorescence in a 96 well plate in your plate reader. By measuring these in your plate reader, you will generate a standard curve of fluorescence for fluorescein concentration. You will be able to use this to convert your cell based readings to an equivalent fluorescein concentration. Before beginning this protocol, ensure that you are familiar with the GFP settings and measurement modes of your instrument. You will need to know what filters your instrument has for measuring GFP, including information about the bandpass width (530 nm / 30 nm bandpass, 25-30nm width is recommended), excitation (485 nm is recommended) and emission (520-530 nm is recommended) of this filter.

Materials:

Fluorescein (provided in kit)
10ml 1xPBS pH 7.4-7.6 (phosphate buffered saline; provided by team)

96 well plate, black with clear flat bottom (provided by team)

Method

Prepare the fluorescein stock solution:

- ❑ Spin down fluorescein kit tube to make sure pellet is at the bottom of tube.
- ❑ Prepare 10x fluorescein stock solution (100 µM) by resuspending fluorescein in 1 mL of 1xPBS. [**Note**: it is important that the fluorescein is properly dissolved. To check this, after the resuspension you should pipette up and down and examine the solution in the pipette tip – if any particulates are visible in the pipette tip continue to mix the solution until they disappear.]
- ❑ Dilute the 10x fluorescein stock solution with 1xPBS to make a 1x fluorescein solution with concentration 10 µM: 100 µL of 10x fluorescein stock into 900 µL 1x PBS

Prepare the serial dilutions of fluorescein:

Accurate pipetting is essential. Serial dilutions will be performed across columns 1-11. COLUMN 12 MUST CONTAIN PBS BUFFER ONLY. Initially you will setup the plate with the fluorescein stock in column 1 and an equal volume of 1xPBS in columns 2 to 12. You will perform a serial dilution by consecutively transferring 100 µl from column to column with good mixing.

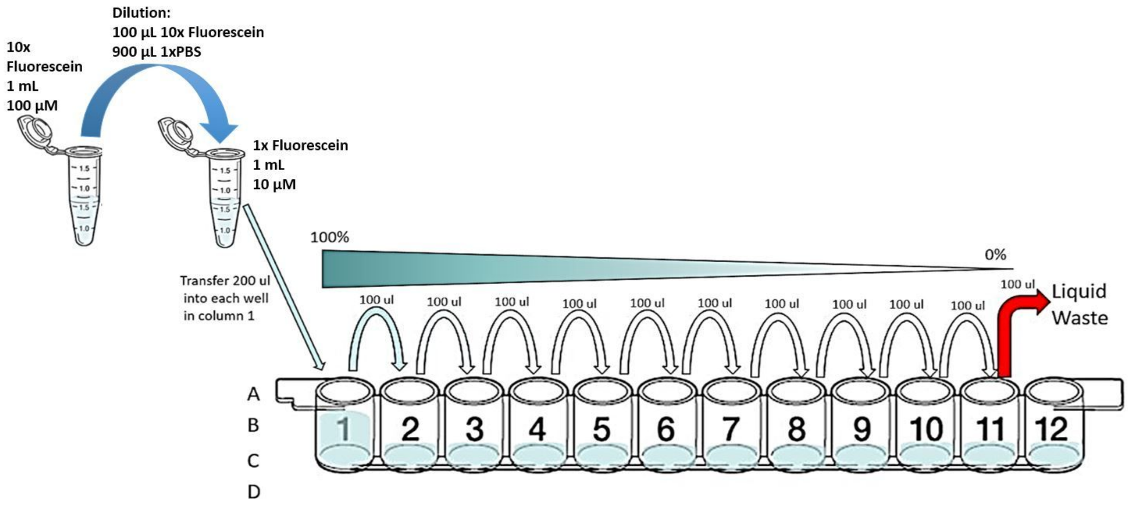

- ❑ **Add 100 µl of PBS** into wells A2, B2, C2, D2....A12, B12, C12, D12
- ❑ **Add 200 µl of fluorescein 1x stock** solution into A1, B1, C1, D1
- ❑ Transfer 100 µl of fluorescein stock solution from A1 into A2.
- ❑ Mix A2 by pipetting up and down 3x and transfer 100 µl into A3…
- ❑ Mix A3 by pipetting up and down 3x and transfer 100 µl into A4...

- ❑ Mix A4 by pipetting up and down 3x and transfer 100 µl into A5...
- ❑ Mix A5 by pipetting up and down 3x and transfer 100 µl into A6...
- ❑ Mix A6 by pipetting up and down 3x and transfer 100 µl into A7...
- ❑ Mix A7 by pipetting up and down 3x and transfer 100 µl into A8...
- ❑ Mix A8 by pipetting up and down 3x and transfer 100 µl into A9...
- ❑ Mix A9 by pipetting up and down 3x and transfer 100 µl into A10...
- ❑ Mix A10 by pipetting up and down 3x and transfer 100 µl into A11...
- ❑ Mix A11 by pipetting up and down 3x and transfer 100 µl into **liquid waste**

TAKE CARE NOT TO CONTINUE SERIAL DILUTION INTO COLUMN 12.

- ❑ Repeat dilution series for rows B, C, D
- ❑ Measure fluorescence of all samples in instrument
- ❑ Record the data in your notebook
- ❑ Import data into Excel sheet provided (**fluorescein standard curve tab**)

Cell measurement protocol

Prior to performing the cell measurements you should perform all three of the calibration measurements. Please do not proceed unless you have completed the three calibration protocols.

Completion of the calibrations will ensure that you understand the measurement process and that you can take the cell measurements under the same conditions. For the sake of consistency and reproducibility, we are requiring all teams to use *E. coli* K-12 DH5-alpha. If you do not have access to this strain, you can request streaks of the transformed devices from another team near you, and this can count as a collaboration as long as it is appropriately documented on both teams' wikis. If you are absolutely unable to obtain the DH5-alpha strain, you may still participate in the InterLab study by contacting the Measurement Committee (measurement at igem dot org) to discuss your situation.

For all of these cell measurements, you must use the same plates and volumes that you used in your calibration protocol. You must also use the same settings (e.g., filters or excitation and emission wavelengths) that you used in your calibration measurements. If you do not use the same plates, volumes, and settings, the measurements will not be valid.

Materials:

Competent cells (*Escherichia coli* strain DH5 α)
LB (Luria Bertani) media
Chloramphenicol (stock concentration 25 mg/mL dissolved in EtOH)
50 ml Falcon tube (or equivalent, preferably amber or covered in foil to block light)
Incubator at 37°C
1.5 ml eppendorf tubes for sample storage
Ice bucket with ice
Micropipettes and tips
96 well plate, black with clear flat bottom preferred (provided by team)

Devices (from Distribution Kit, all in pSB1C3 backbone):

**Device**	**Part Number**	**Plate**	**Location**
Negative control	BBa_R0040	Kit Plate 7	Well 2D
Positive control	BBa_I20270	Kit Plate 7	Well 2B
Test Device 1	BBa_J364000	Kit Plate 7	Well 2F
Test Device 2	BBa_J364001	Kit Plate 7	Well 2H
Test Device 3	BBa_J364002	Kit Plate 7	Well 2J
Test Device 4	BBa_J364007	Kit Plate 7	Well 2L
Test Device 5	BBa_J364008	Kit Plate 7	Well 2N
Test Device 6	BBa_J364009	Kit Plate 7	Well 2P

Workflow

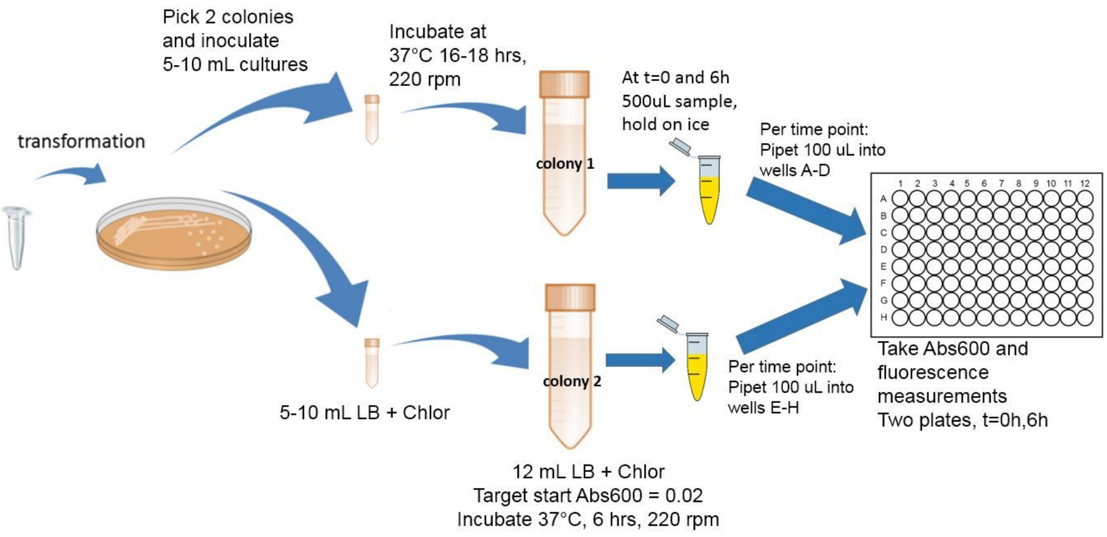

Method

Day 1: transform *Escherichia coli* DH5 α with these following plasmids (all in pSB1C3):

**Device**	**Part Number**	**Plate**	**Location**
Negative control	BBa_R0040	Kit Plate 7	Well 2D
Positive control	BBa_I20270	Kit Plate 7	Well 2B
Test Device 1	BBa_J364000	Kit Plate 7	Well 2F
Test Device 2	BBa_J364001	Kit Plate 7	Well 2H
Test Device 3	BBa_J364002	Kit Plate 7	Well 2J
Test Device 4	BBa_J364007	Kit Plate 7	Well 2L
Test Device 5	BBa_J364008	Kit Plate 7	Well 2N
Test Device 6	BBa_J364009	Kit Plate 7	Well 2P

Help Debugging Your Transformations:

- We STRONGLY recommend that you use the iGEM protocol to create your competent cells: http://parts.igem.org/Help:Protocols/Competent_Cells
- Once you have created your competent cells, we STRONGLY recommend that you measure the competency of your cells using the Competent Cell Test Kit: http://parts.igem.org/Help:2017_Competent_Cell_Test_Kit
- Finally, we STRONGLY recommend that you closely follow the iGEM protocols for resuspending DNA from the kit plates and performing the transformation: http://parts.igem.org/Help:Protocols/Transformation

Year after year, we have found that most teams are highly successful when they follow these protocols, even if alternative protocols are used within your lab. If you are having trouble transforming your test devices, please try the protocols above.

Day 2: Pick 2 colonies from each of the transformation plates and inoculate in 5-10 mL LB medium + Chloramphenicol. Grow the cells overnight (16-18 hours) at 37°C and 220 rpm.

Day 3: Cell growth, sampling, and assay

- ☐ Make a 1:10 dilution of each overnight culture in LB+Chloramphenicol (0.5mL of culture into 4.5mL of LB+Chlor)
- ☐ Measure Abs_{600} of these 1:10 diluted cultures
- ☐ Record the data in your notebook
- ☐ Dilute the cultures further to a target Abs_{600} of 0.02 in a final volume of **12 ml** LB medium + Chloramphenicol in 50 mL falcon tube (amber, or covered with foil to block light).
- ☐ Take 500 µL samples of the diluted cultures at 0 hours into 1.5 ml eppendorf tubes, prior to incubation. (At each time point 0 hours and 6 hours, you will take a sample from each of the 8 devices, two colonies per device, for a total of 16 eppendorf tubes with 500 µL samples per time point, 32 samples total). Place the samples on ice.
- ☐ Incubate the remainder of the cultures at 37°C and 220 rpm for 6 hours.
- ☐ Take 500 µL samples of the cultures at 6 hours of incubation into 1.5 ml eppendorf tubes. Place samples on ice.
- ☐ At the end of sampling point you need to measure your samples (Abs_{600} and fluorescence measurement), see the below for details.
- ☐ Record data in your notebook
- ☐ Import data into Excel sheet provided (**fluorescence measurement tab**)

Measurement

Samples should be laid out according to the plate diagram below. Pipette 100 µl of each sample into each well. From 500 µl samples in a 1.5 ml eppendorf tube, 4 replicate samples of colony #1 should be pipetted into wells in rows A, B, C and D. Replicate samples of colony #2 should be pipetted into wells in rows E, F, G and H. Be sure to include 8 control wells containing 100uL each of only LB+chloramphenicol on each plate in column 9, as shown in the diagram below. Set the instrument settings as those that gave the best results in your calibration curves (no measurements off scale). If necessary you can test more than one of the previously calibrated settings to get the best data (no measurements off scale). Instrument temperature should be set to room temperature (approximately 20-25 C) if your instrument has variable temperature settings.

Help Debugging:

- If you have measurements that are off scale ("OVERFLOW"), that data will not be usable. You need to adjust your settings so that the data will be in range and re-run your calibration.
- If your Abs600 measurements for your cell colonies are very close to that of your LB+Chlor, then your cells have probably not been transformed correctly or grown correctly.
- If your negative and positive control values are very close to each other, that probably means something has gone wrong in your protocol or measurement.

Layout for Abs_{600} and Fluorescence measurement

At the end of the experiment, you should have two plates to read. Each plate should be set up as shown below. You will have one plate for each time point: 0 and 6 hours. On each plate you will read both fluorescence and absorbance.

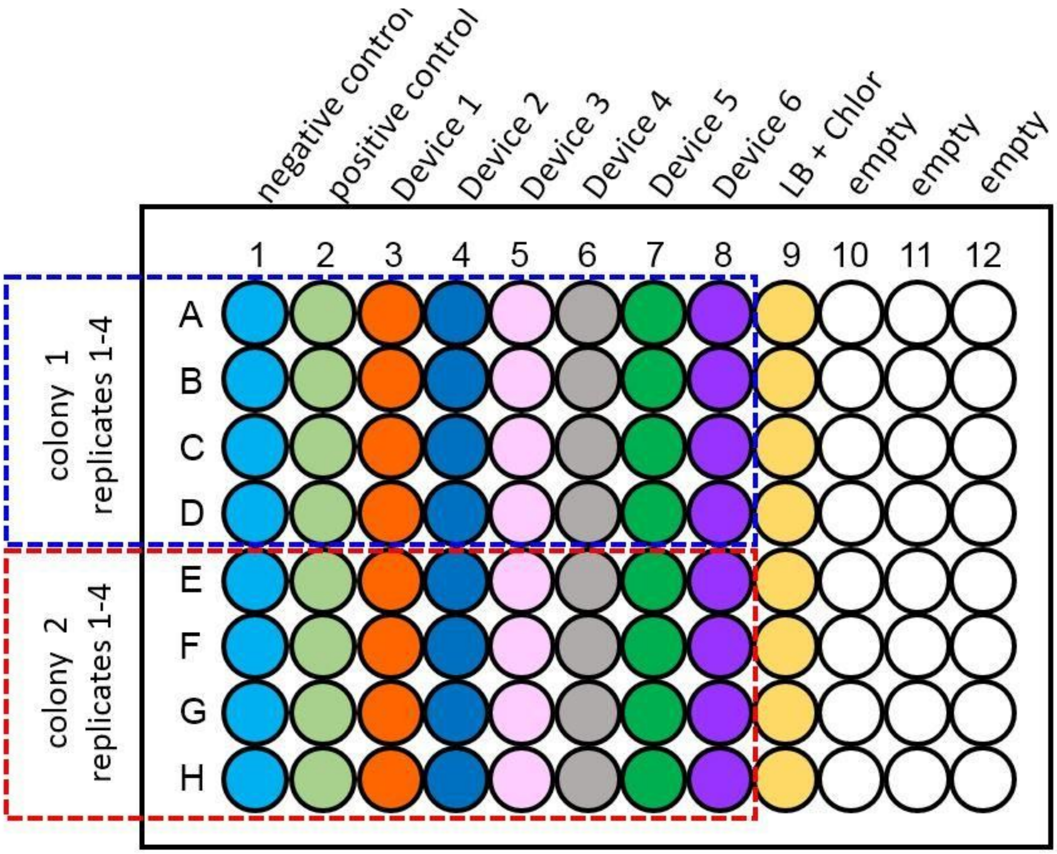

Protocol: Colony Forming Units per 0.1 OD_{600} *E. coli* cultures

This procedure can be used to calibrate OD_{600} to colony forming unit (CFU) counts, which are directly relatable to the cell concentration of the culture, i.e. viable cell counts per mL. This protocol assumes that 1 bacterial cell will give rise to 1 colony.

For the CFU protocol, you will need to count colonies for your two Positive Control (BBa_I20270) cultures and your two Negative Control (BBa_R0040) cultures.

Step 1: Starting Sample Preparation
This protocol will result in CFU/mL for 0.1 OD_{600}. Your overnight cultures will have a much higher OD_{600} and so this section of the protocol, called "Starting Sample Preparation", will give you the "Starting Sample" with a 0.1 OD_{600} measurement.

1. Measure the OD_{600} of your cell cultures, making sure to dilute to the linear detection range of your plate reader, e.g. to 0.05 – 0.5 OD_{600} range. Include blank media (LB + Cam) as well.

   For an overnight culture (16-18 hours of growth), we recommend diluting your culture 1:8 (8-fold dilution) in LB + Cam before measuring the OD_{600}.

   Preparation: Add 25 µL culture to 175 µL LB + Cam in a well in a black 96-well plate, with a clear, flat bottom.

   Recommended plate setup is below. Each well should have 200 µL .

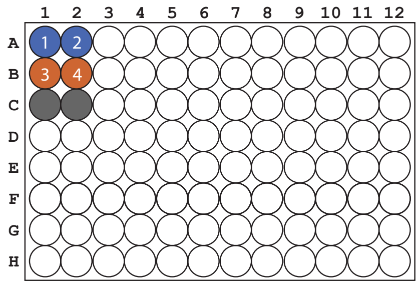

A1 - A2: Positive Controls (cultures 1-2)
B1 - B2: Negative Controls (cultures 3-4)
C1 - C2: Blank media - 200 µL of LB + Cam (in duplicate)

2. Dilute your overnight culture to OD_{600} = 0.1 in 1mL of LB + Cam media. Do this in triplicate for each culture.

   Use $(C_1)(V_1) = (C_2)(V_2)$ to calculate your dilutions
   C_1 is your starting OD_{600} — C_2 is your target OD_{600} of 0.1
   V_1 is the unknown volume in µL — V_2 is the final volume of 1000 µL

   Important: When calculating C_1, subtract the blank from your reading and multiple by the dilution factor you used.
   Example: C1 = (1:8 OD_{600} - blank OD_{600}) x 8 = (0.195 - 0.042) x 8 = 0.153 x 8 = 1.224

   Example: $(C_1)(V_1) = (C_2)(V_2)$
   (1.224)(x) = (0.1)(1000µL)
   x = 100/1.224 = 82 µL culture
   Add 82 µL of culture to 918 µL media for a total volume of 1000 µL

3. Check the OD_{600} and make sure it is 0.1 (minus the blank measurement).
   Recommended plate setup is below. Each well should have 200 µL .

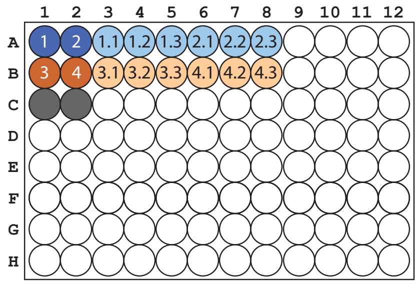

A1 - A2: Positive Controls (cultures 1-2)
B1 - B2: Negative Controls (cultures 3-4)
C1 - C2: Blank media - 200 µL of LB + Cam (in duplicate)
A3 - A8: 0.1 Starting Sample Dilutions for Positive Controls (in triplicate per culture, 6 total dilutions)
B3 - B8: 0.1 Starting Samples Dilutions for Negative Controls (in triplicate per culture, 6 total dilutions)

Step 2: Dilution Series Instructions

Do the following serial dilutions for your triplicate Starting Samples you prepared in Step 1. You should have 12 total Starting Samples - 6 for your Positive Controls and 6 for your Negative Controls.

For each Starting Sample (*total for all 12 showed in italics in paraenthesis*):

1. You will need 3 LB Agar + Cam plates (*36 total*).
2. Prepare three 2.0 mL tubes (*36 total*) with 1900 µL of LB + Cam media for Dilutions 1, 2, and 3 (see figure below).
3. Prepare two 1.5 mL tubes (*24 total*) with 900 µL of LB + Cam media for Dilutions 4 and 5 (see figure below)..
4. Label each tube according to the figure below (Dilution 1, etc.) for each Starting Sample.
5. Pipet 100 µL of Starting Culture into Dilution 1. Discard tip. Do NOT pipette up and down. Vortex tube for 5-10 secs.
6. Repeat Step 5 for each dilution through to Dilution 5 as shown below.
7. Aseptically spead plate 100 µL on LB + Cam plates for Dilutions 3, 4, and 5.
8. Incubate at 37°C overnight and count colonies after 18-20 hours of growth.

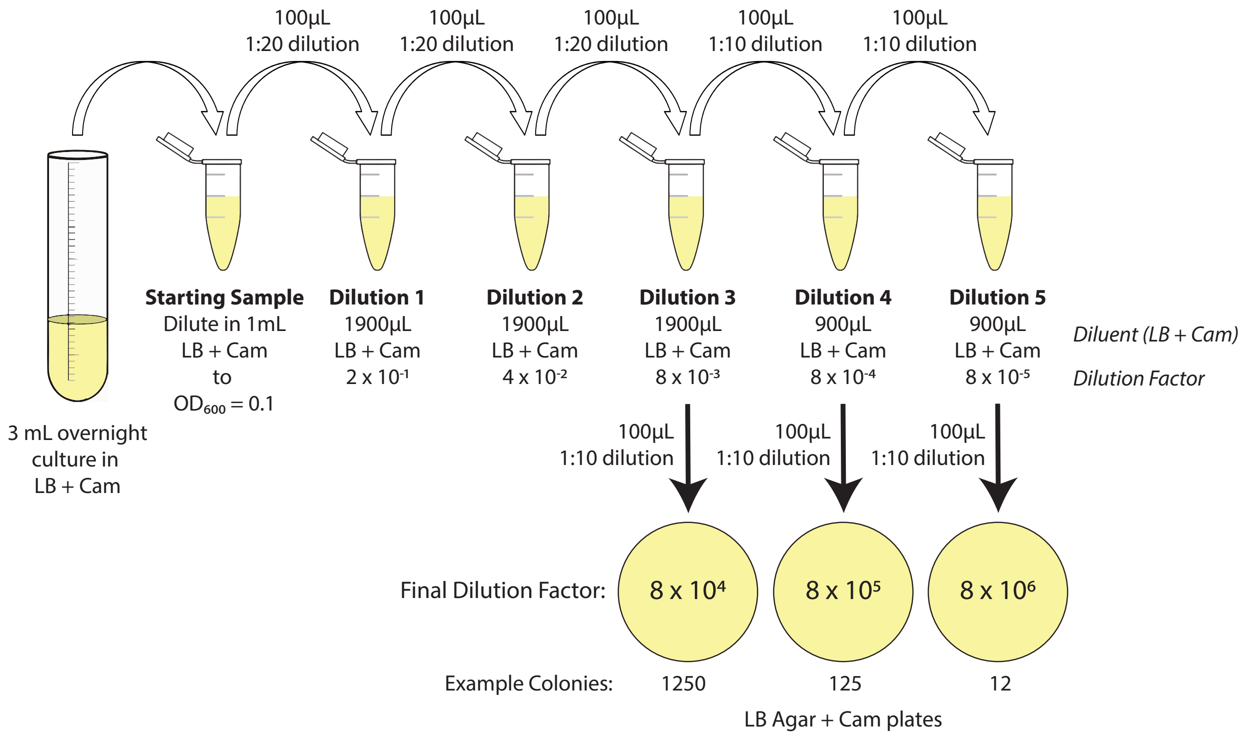

Step 3: CFU/mL/OD Calculation Instructions

Based on the assumption that 1 bacterial cell gives rise to 1 colony, colony forming units (CFU) per 1mL of an OD_{600} = 0.1 culture can be calculated as follows:

1. Count the colonies on each plate with fewer than 300 colonies.
2. Multiple the colony count by the Final Dilution Factor on each plate.

Example using Dilution 4 from above

# colonies	x	Final Dilution Factor	= CFU/mL
125	x	(8×10^5)	$= 1 \times 10^8$ CFU/mL in Starting Sample (OD_{600} = 0.1)

Edit this form

2016 InterLab Worksheet for Flow Cytometry

Every team that participates in the 2016 iGEM InterLab study needs to fill in this form.

IMPORTANT: If you measured your devices using multiple pieces of equipment, please fill out this form for each different type of measurement you obtained.

If you have any questions or problems filling in this form, please email us at measurement at igem dot org.

* Required

Team name *

Please enter your team name below.

InterLab Study wiki page *

Please provide the direct link to your team's wiki page for the InterLab study. As explained in the InterLab requirements, all teams must have a specific page for the InterLab study on their wiki.

Individuals responsible for conducting InterLab study *

Please list everyone involved with creating the devices, measuring them, and processing the data. Please indicate which role each person filled. List anyone else who should be credited, e.g., in a publication based on this data.

Date of InterLab Study *

Please note the date(s) that the measurement was obtained.

Did your team participate in the Extra Credit? *

Details for the Extra Credit in 2015 can be found here:
http://2016.igem.org/Tracks/Measurement/Interlab_study

- ◯ Yes
- ◯ No

If you participated in Extra Credit, please enter a link to a page on your team wiki that describes your extra credit work *

Equipment Information

What type of incubator did you use to grow your cells? *

Please provide as much information as possible in terms of the equipment type, name, and model.

If known, what was your incubator's throw (shaking diameter)?

This information is often found with the manufacturer's specifications for the incubator.

What model of flow cytometer did you use to measure the devices? *

Please provide as much information as possible in terms of the equipment type, name, model, and any custom modifications that have been made to the instrument.

When was this equipment last calibrated? *

Who calibrated the equipment? *

What was the wavelength of light you used to excite the cells? *

The recommended wavelength for GFP is a 488nm laser

What was the filter on the channel you used to capture the light emission from the cells? *

The recommended filter for GFP is 530/30 (meaning a bandpass filter with 530 nm center, 30 nm width)

What quantity was recorded for each event? *

Flow cytometers may report “area”, “height” and/or “width” of each event. If possible, you should report area, which will often be indicated by a “-A” in the channel name, e.g., “FITC-A.” The reason to favor area is that it gives the total fluorescence observed for each particle, whereas height gives maximum fluorescent intensity and width gives the length the particle was observed.

- Area
- Height
- Width
- Other:

If you have other information or details you wish to provide about your equipment, please use the box below.

Prepare Cells for Flow Cytometry

Day 1 : Transformation (or streak plate) *

Transform Escherichia coli DH5α or TOP10 with these following plasmids (each plasmid in a different sample). Please check off each step that you followed. If you did anything differently or extra, please note that in the "Other" box. Likewise, if you have already transformed and are streaking a fresh plate for colonies, note that in the "Other" box.

- Positive control
- Negative control
- Device 1: J23101+I13504
- Device 2: J23106+I13504
- Device 3: J23117+I13504
- Other:

Day 2 : Cell growth *

Please check off each step that you followed. If you did anything differently or extra, please note that in the "Other" box.

- Pick 3 colonies from each of plate and inoculate it on 5-10 mL LB medium + Chloramphenicol (For antibiotic concentrations, please follow these guidelines: http://parts.igem.org/Help:Protocols/Antibiotic_Stocks).
- Grow the cells overnight (16-18 hours) at 37°C and 220 rpm.

- [ ] Other:

Continue: Did you set up biological replicates in triplicate? *

Biological replicates are where different samples that are expected to be identical are measured. This informs you about the variability across your organisms that contain the same device. For example, if you are using E. coli, this would be done by measuring the fluorescence from 3 different colonies containing the same device.

- [ ] Yes
- [ ] No

Day 3 : Cell growth, sampling, and assay *

Please check off each step that you followed. If you did anything differently or extra, please note that in the "Other" box. We recommend you read the OD600 of your culture, if possible. Please note the instrument you used to measure OD600 in the "Other" box if you were able to do so.

- [ ] Set your instrument to read OD600 (as OD calibration setting)
- [ ] Measure OD600 of the overnight cultures
- [ ] Dilute the cultures to a target OD600 of 0.02 (see the volume of preloading culture and media in Excel (normalisation) sheets) in 10 ml 0.5x TB medium + Chloramphenicol in 50 mL falcon tube (if using cuvettes, you can use 100 ml in a 500 ml shake flask).
- [ ] Incubate the cultures at 37°C and 220 rpm.
- [ ] Take 100 µL (1% of total volume) cultures at 6 hours of incubation (if using cuvettes, remove 1 ml from 100 ml culture).
- [ ] Dilute cells to the appropriate density for your flow cytometer. If unknown, check with the person who maintains the instrument.
- [ ] Measure cells using flow cytometer.
- [ ] Other:

What is the initial OD600 measurement of your overnight cultures? *

Please list all devices and replicate number (if applicable) with their OD600 next to them.

What type of media did you use for this step? *

For E. coli, we recommend Terrific broth (at half strength: 0.5x TB) or can use LB (Luria Bertani) media as an alternative supplemented with the appropriate antibiotic. For antibiotic concentrations, please follow these guidelines: http://parts.igem.org/Help:Protocols/Antibiotic_Stocks

- ( ) 0.5x Terrific Broth
- ( ) Luria Bertani
- ( ) Other:

What type of vessel or container did you use to grow your cells? *

- ( ) 10 ml culture tube
- ( ) 50 ml Falcon tube

- ◯ 500 ml shake flask
- ◯ Other: ____

Acquire Data with Flow Cytometer

Please check off each step that you followed. If you did anything differently or extra, please note that in the "Other" box. *

- ☐ Adjust side-scatter (SSC) and forward scatter (FSC) PMT voltages using bacteria from your negative control, until the distribution of each is centered on the scale.
- ☐ Adjust FITC/GFP PMT voltage using bacteria from your positive control, until the upper edge of the "bell curve" from the fluorescent population is one order of magnitude below the upper end of the scale.
- ☐ Acquire at least 10,000 events from a sample of calibration beads.
- ☐ Acquire at least 10,000 events for each biological sample.
- ☐ Other: ____

Calibration to Standard Units

Please check off each step that you followed. If you did anything differently or extra, please note that in the "Other" box. If you have followed the rest of this protocol, you should be able to use the "flow_cyometry_workbook" Excel file to make the unit conversion calculations. *

- ☐ Examine a histogram of the RCP-30-5A beads and find the observed centers of at least the three most strongly fluorescent large peaks. See the provided example file.
- ☐ Divide the MEFL value for each RCP-30-5A peak by the observed peak centers, to produce a conversion ratio.
- ☐ Take the average of the conversion ratios: multiplying arbitrary units by this mean conversion ratio will change them into MEFL.
- ☐ Compute the geometric mean of fluorescence for each biological sample, excluding all events with values below 10.
- ☐ Multiply the geometric mean fluorescence for each sample by the mean conversion ratio, to produce a value in MEFL.
- ☐ Other: ____

Report Results

Please fill in the provided Excel file and email it to measurement@igem.org. If you cannot use the Excel file, please use another spreadsheet program or email the measurement@igem.org to arrange how best to contribute your data.

Feedback

Please rate your experience with conducting the InterLab Study *

- ◯ Very easy to participate; little to no problems
- ◯ Cloning problems made it more difficult than expected
- ◯ Equipment problems made it more difficult than expected
- ◯ Instructions were unclear and made it difficult to participate

- ◯ Very difficult to participate; numerous problems
- ◯ Other: [ ]

Please rate your experience with filling in this InterLab Worksheet *

- ◯ Very easy to fill in, no problems
- ◯ Took a long time to fill out, but was easy to understand
- ◯ Did not understand one or two questions
- ◯ Did not understand an entire section
- ◯ Very difficult to use; numerous problems
- ◯ Other: [ ]

Please let us know any other thoughts or comments you have about the InterLab study experience.

Submit

Never submit passwords through Google Forms.

100%: You made it.

Powered by Google Forms

This content is neither created nor endorsed by Google.

Report Abuse - Terms of Service - Additional Terms

Extra Credit: Flow Cytometry

For extra credit, teams with access to a flow cytometer and SpheroTech calibration beads can collect and submit flow cytometry data as well. Teams performing this additional measurement will be given special acknowledgement at the iGEM Jamboree and in any resulting scientific publications.

Materials:
SpheroTech Rainbow calibration beads, type RCP-30-5A or URCP-38-2K
(http://www.spherotech.com/CalibrationParticles.htm)

Record the lot number for your calibration beads.
It should be one or two letters followed by a number (e.g., "AJ02")

Method:
During your cell measurement protocol, prepare a sample of SpheroTech beads according to the manufacturer instructions and place in well A10 of each plate.

Measurement:
After measuring each plate with your plate reader, also collect data from all wells using your flow cytometer. Follow your flow cytometer instructions for collecting samples and dilute further if necessary. Collect at least 10,000 events per well.

On the interlab form, mark that you have done the flow cytometry extra credit, and enter your instrument information in the fields provided.

Name the FCS files for your experimental samples following these templates:

- Cell samples: [team]_[time]h_[well]_[construct].fcs
  example: WPI_6h_A1_NegativeControl.fcs
- Blanks: [team]_[time]h_[well]_Blank.fcs
  example: WPI_6h_A9_Blank.fcs
- Beads: [team]_[time]h_[well]_[type]_[lot].fcs
  example: WPI_6h_A10_URCP-38-2K_AJ02.fcs

Bundle all FCS files together into a zip or tar file and upload to DropBox at:
http://2018.igem.org/Measurement/InterLab/Flow_Cytometry

If you cannot access DropBox, email the measurement committee to make alternate arrangements for delivering your files.
