# Supplementary material for: Comparative analysis of three studies measuring fluorescence from engineered bacterial genetic constructs
Source: PLoS One. 2021 Jun 7;16(6):e0252263. doi: 10.1371/journal.pone.0252263 (PMC8183995; doi:10.1371/journal.pone.0252263)
Supplement: S4 File — Quality control criteria for inclusion of data for each team. (PDF) [file pone.0252263.s004.pdf]

# S3 File Data Acceptance Criteria

Jacob Beal, Geoff S. Baldwin, Natalie G. Farny, Markus Gershater,
Traci Haddock-Angelli, Russell Buckley-Taylor, Ari Dwijayanti, Daisuke Kiga,
Meagan Lizarazo, John Marken, Kim de Mora, Randy Rettberg,
Vishal Sanchania, Vinoo Selvarajah, Abigail Sison, Marko Storch,
Christopher T. Workman, and the iGEM Interlab Study Contributors

Each data set used in each year of the interlaboratory study was evaluated against the following criteria to determine whether it was of sufficient quality for inclusion. These criteria were not intended to be stringent, but rather to represent a minimal "sanity check" against major errors in protocol execution or reporting.

For 2016, criteria are applied retrospectively to remove likely-flawed datasets from analysis, resulting in a total collection of 36 accepted datasets out of 65; the number of datasets passing for each criteria is detailed in Figure 1. In 2017 and 2018, all criteria were applied before acceptance of data from teams, and any teams whose data did not meet all of these criteria were invited to re-execute the experiment in order to correct the deficiencies in their data. For 2018, similar criteria were also applied to additional calibrant measurements, which are not analyzed in this manuscript because they were not included in prior years and have already been presented in [1].

The criteria are:

- Water measurements have a lower OD than LUDOX measurements.
- Water OD measurements are not negative.
- Fluorescein fluorescence measurements generally decreases with increasing dilution (excepting saturation)
- PBS-only fluorescence measurements are not negative.
- Cell sample fluorescence measurements are within the range covered by fluorescein samples.
- Positive control is brighter than negative control at 6 hours and also greater than zero
- At least half of cell sample ODs increase significantly from 0 hours to 6 hours (i.e., cells are generally alive and growing)
- Fluorescence/OD measurements for test constructs at 6 hours span at least a 10-fold range (i.e., there is at least some significant variability in fluorescence expression)
- All replicates are present for every sample.

## References

[1] Beal J, Farny NG, Haddock-Angelli T, Selvarajah V, Baldwin GS, Buckley-Taylor R, et al. Robust estimation of bacterial cell count from optical density. Communications biology. 2020;3(1):1–29.

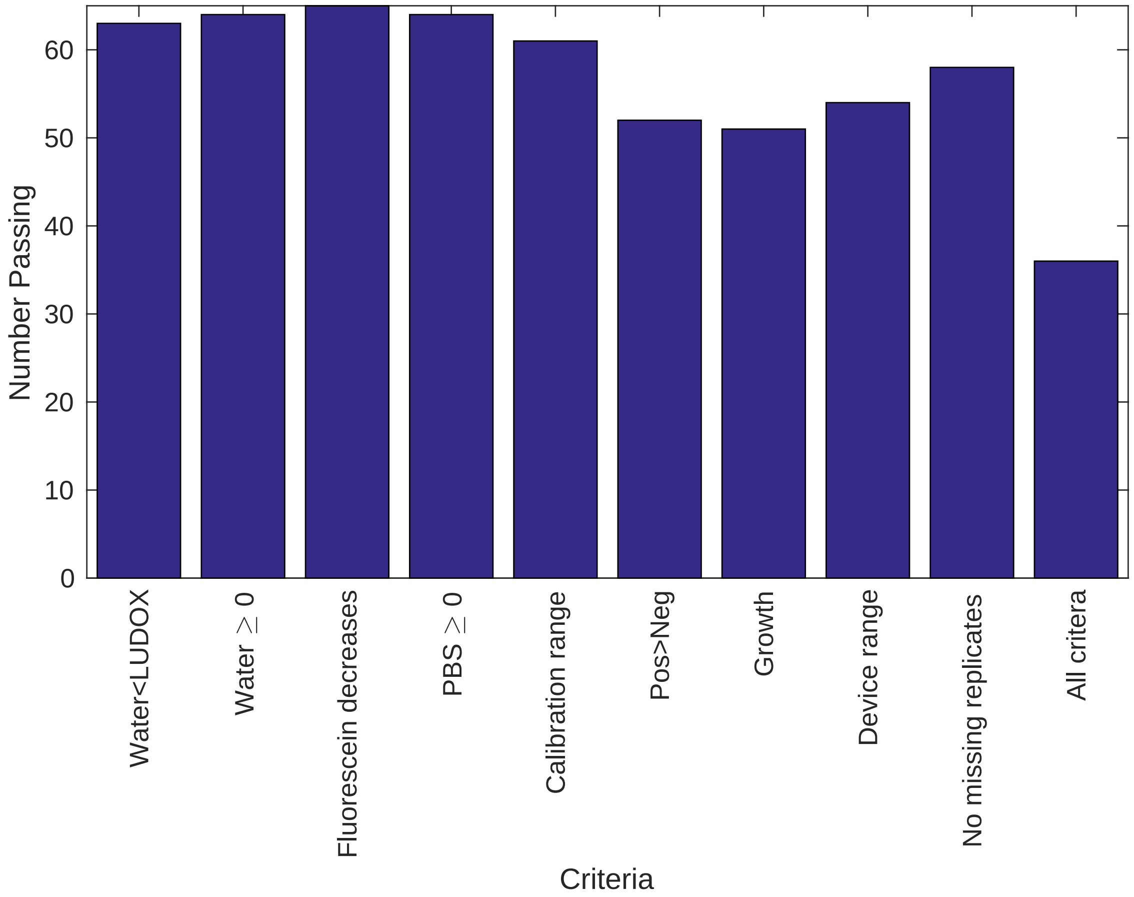


Figure 1: Number of data sets from 2016 passing 2017/2018 acceptance criteria.
