# Supplementary material for: Comparative analysis of three studies measuring fluorescence from engineered bacterial genetic constructs
Source: PLoS One. 2021 Jun 7;16(6):e0252263. doi: 10.1371/journal.pone.0252263 (PMC8183995; doi:10.1371/journal.pone.0252263)
Supplement: S5 File — Details of how flow cytometry data processing was conducted for 2018 data. (PDF) [file pone.0252263.s005.pdf]

S4 File Flow Cytometry Data Processing for 2018

Jacob Beal, Geoff S. Baldwin, Natalie G. Farny, Markus Gershater, Traci Haddock-Angelli, Russell Buckley-Taylor, Ari Dwijayanti, Daisuke Kiga, Meagan Lizarazo, John Marken, Kim de Mora, Randy Rettberg, Vishal Sanchania, Vinoo Selvarajah, Abigail Sison, Marko Storch, Christopher T. Workman, and the iGEM Interlab Study Contributors

With the flow cytometry data for the 2018 study, a unit conversion model from arbitrary units to MEFL was constructed per the recommended best practices of TASBE Flow Analytics for each data set using the bead sample and lot information provided by each team:

- Gating was automatically determined using a two-dimensional Gaussian fit on the forward-scatter area and side-scatter area channels for the first negative control. An example is shown in Figure 1.
- The same negative control was used to determine autofluorescence for background subtraction. An example is shown in Figure 2.
- As only a single green fluorescent protein was used, there was no need for spectral compensation or color translation.
- All teams submitting flow cytometry used standard SpheroTech Rainbow Calibration beads [1] for dye-based calibration to equivalent fluorescent molecules [2]. In particular, 16 teams used RCP-30-5A beads (various lot numbers) and 1 team used URCP-38-2K beads, and conversion from arbitrary units to MEFL was computed using the peak-to-intensity values provided for each lot. Examples are provided in Figure 3 and Figure 4.

The color model thus produced for each data set was then applied to each sample in that data set to filter events and convert GFP measurements from arbitrary units to MEFL, and geometric mean and standard deviation computed for the filtered collection of events.

References

[1] SpheroTech. Measuring Molecules of Equivalent Fluorescein (MEFL), PE (MEPE) and RPE-CY5 (MEPCY) using Sphero Rainbow Calibration Particles. SpheroTech; 2001. SpheroTechnical Notes: STN-9, Rev C 071398.

[2] Hoffman RA, Wang L, Bigos M, Nolan JP. NIST/ISAC standardization study: Variability in assignment of intensity values to fluorescence standard beads and in cross calibration of standard beads to hard dyed beads. Cytometry Part A. 2012;81(9):785–796.

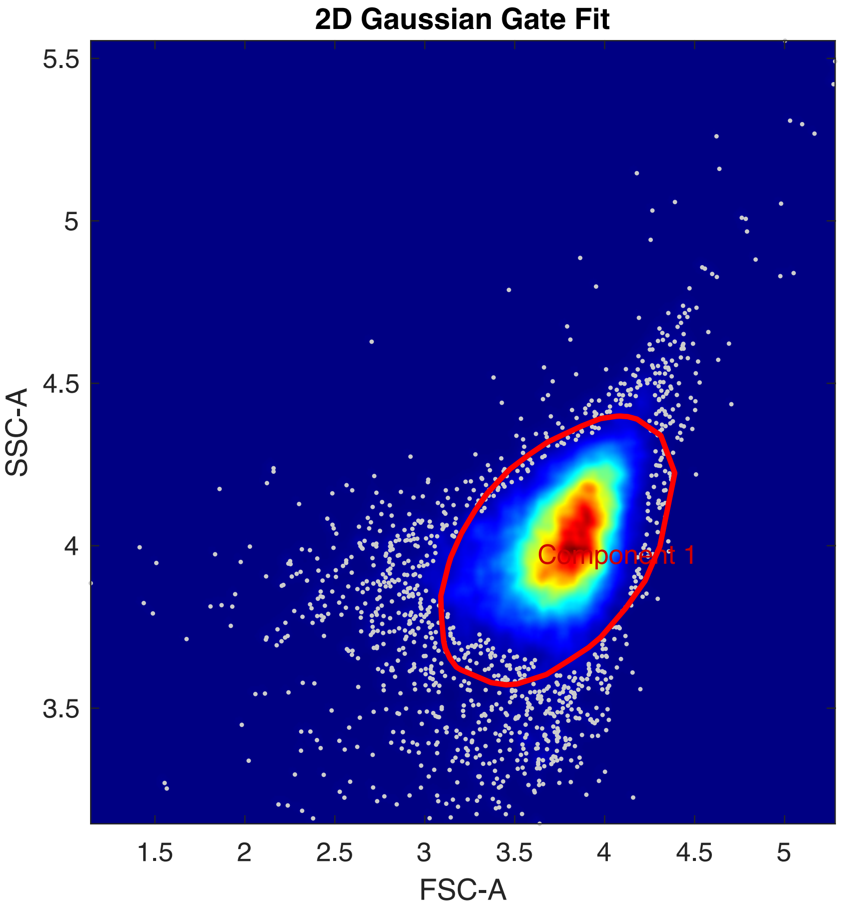

Figure 1: Prototypical example of two-dimensional Gaussian fit used for determination of gating from negative control.

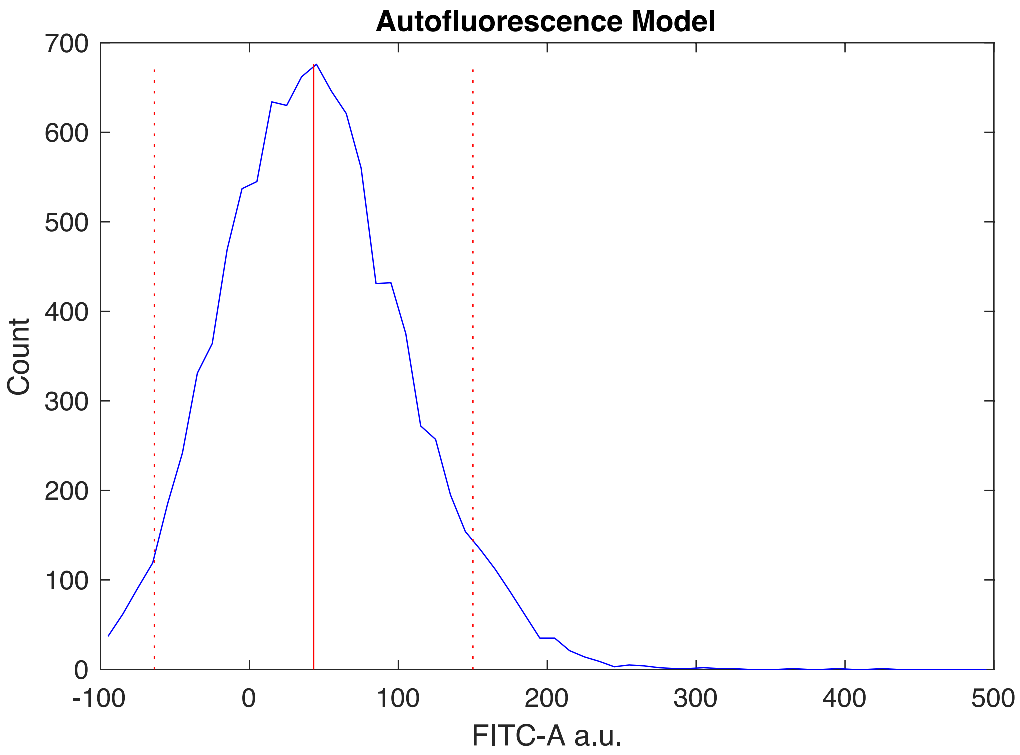

Figure 2: Prototypical example of mean and standard deviation computation of autofluorescence from from negative control.

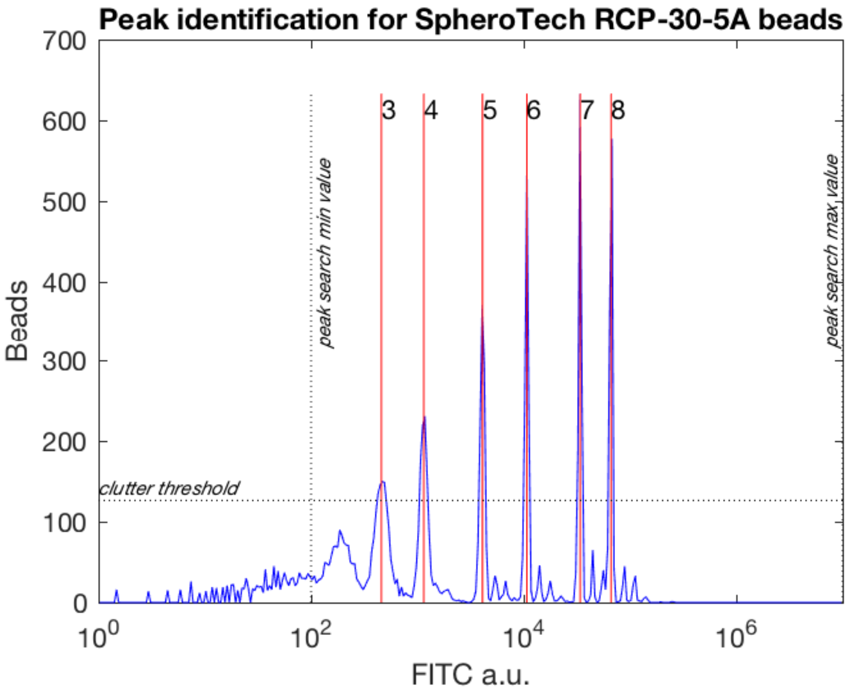

Figure 3: Prototypical example of calibration bead sample showing peak detection across a broad linear range.

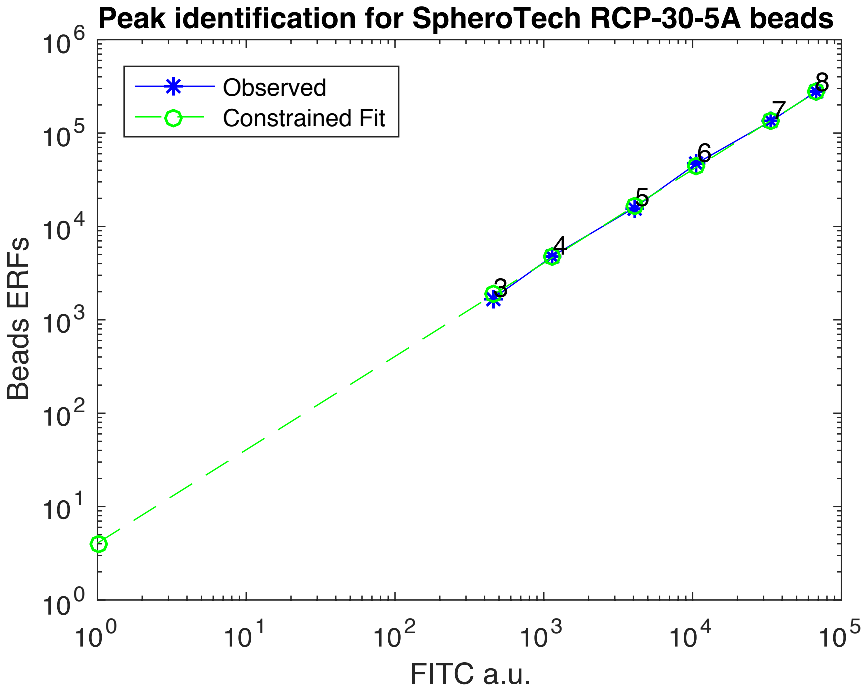

Figure 4: Prototypical example of unit conversion from arbitrary units to MEFL computed from calibration bead peaks.
