# Supplementary figures and images for: Comparative analysis of three studies measuring fluorescence from engineered bacterial genetic constructs
Source: PLoS One. 2021 Jun 7;16(6):e0252263. doi: 10.1371/journal.pone.0252263 (PMC8183995; doi:10.1371/journal.pone.0252263)

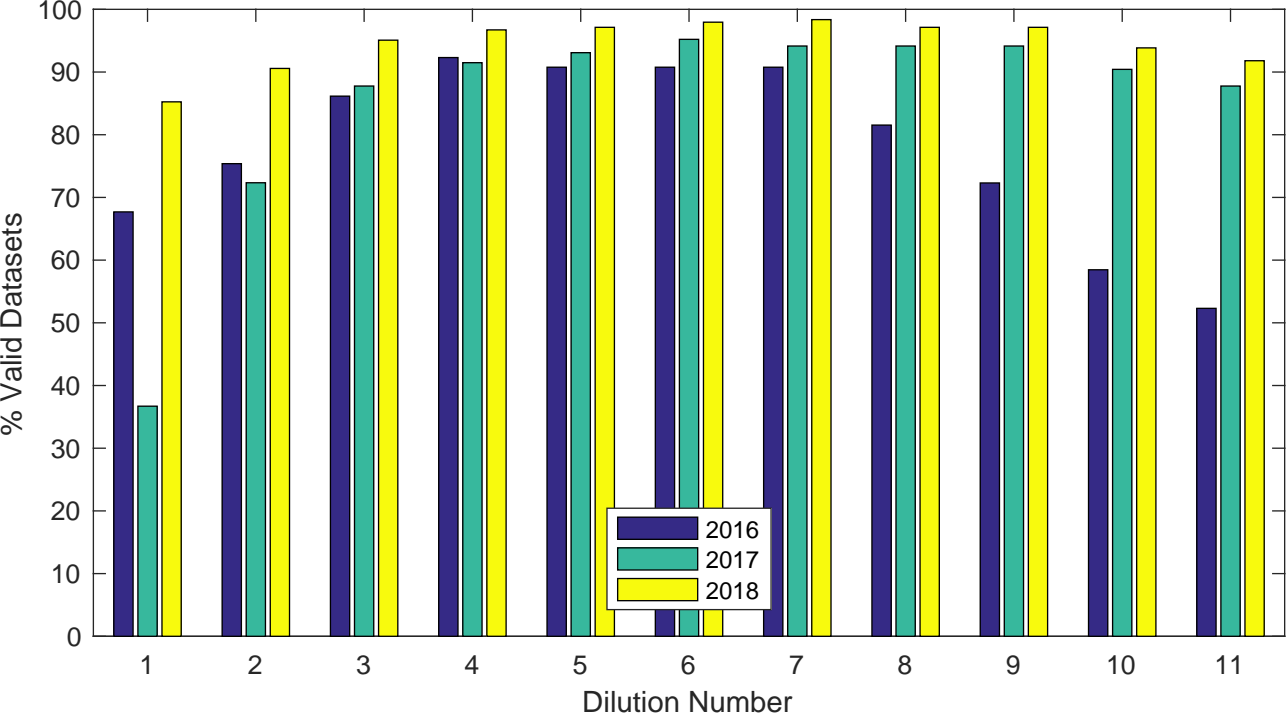

Supplement: S1 Fig — Fraction of data sets with valid fluorescein dilution levels for each dilution number in the series. (PDF) [file pone.0252263.s008.pdf]
