# Supplementary material for: Comparative analysis of three studies measuring fluorescence from engineered bacterial genetic constructs
Source: PLoS One. 2021 Jun 7;16(6):e0252263. doi: 10.1371/journal.pone.0252263 (PMC8183995; doi:10.1371/journal.pone.0252263)
Supplement: S2 Fig — Replicate measurements of fluorescent calibrants. (PDF) [file pone.0252263.s009.pdf]

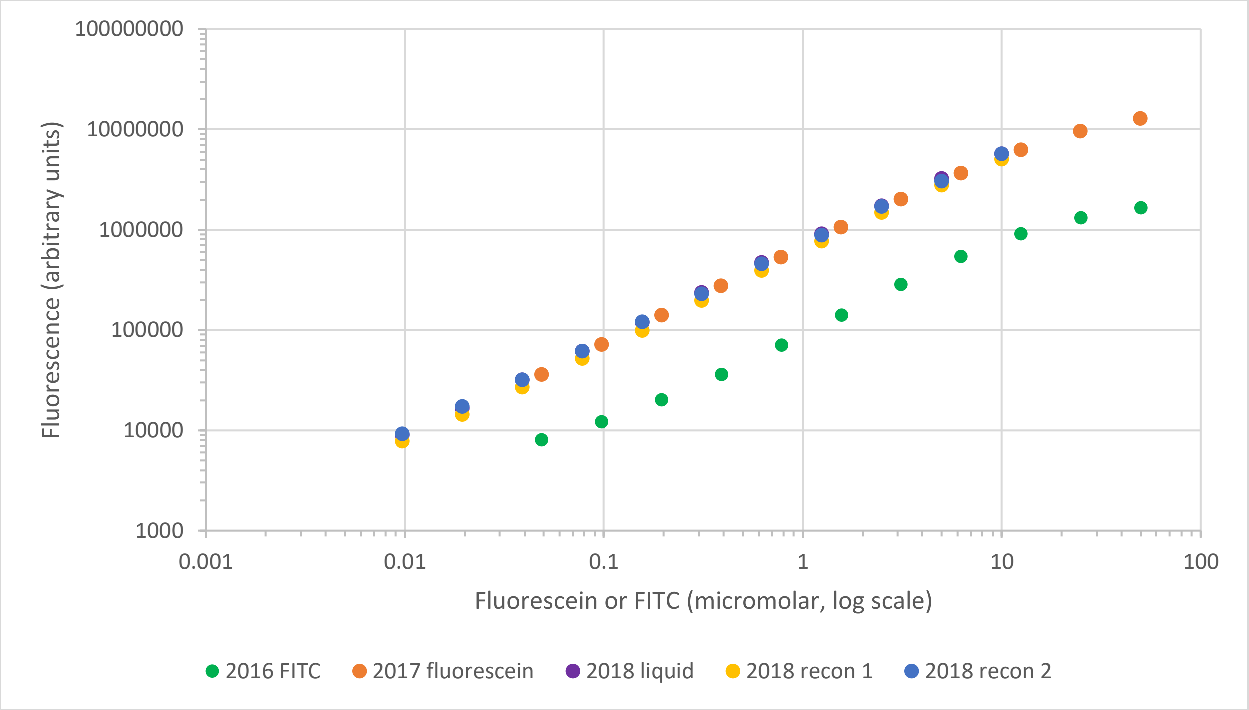

Figure S2: Replicate measurements of fluorescent calibrants. All calibrants were prepared, and dilutions in quadruplicate performed, according to the corresponding Interlab Study Protocols for that year (see supplementary files for protocols). In 2018, the recommended starting concentration for the dilution series was 10µM, so higher concentrations were not measured. The dilutions used to create the data are as indicated on the sample tube and according to the relevant protocol for that year. The samples 2016 FITC (green), 2017 fluorescein (orange), 2018 recon 1 (yellow) and 2018 recon 2 (blue) were reconstituted according to the study protocols. The sample 2018 liquid (purple) is an aliquot of the original 2018 calibrant stock obtained prior to drying for distribution, with values nearly identical to 2018 recon 2. All samples were read on the same PerkinElmer Wallac Victor3 Multilabel Plate Reader at excitation 485nm and emission 535nm.
