# Supplementary material for: Comparative analysis of three studies measuring fluorescence from engineered bacterial genetic constructs
Source: PLoS One. 2021 Jun 7;16(6):e0252263. doi: 10.1371/journal.pone.0252263 (PMC8183995; doi:10.1371/journal.pone.0252263)
Supplement: Attachment — Submitted filename: R2R.docx [file pone.0252263.s010.docx]

**Editorial Requests:**

*1. Please ensure that your manuscript meets PLOS ONE's style requirements, including those for file*

*naming.*

To the best of our knowledge, the manuscript meets all style requirements. If there are problems, please

let us know specifics and we will correct them.

*2. Thank you for stating the following in the Financial Disclosure section:*

*"Partial support for this work was provided by NSF Expeditions in Computing Program 469 Award*

*#1522074 as part of the Living Computing Project. Funder URL: https://www.nsf.gov/*

*The funders had no role in study design, data collection and analysis, decision to publish, or preparation*

*of the manuscript."*

*And competing Interest:*

*"The authors of this manuscript have read the journal’s policy and have the following competing*

*interests: The authors received no specific commercial funding for this work. The following authors are*

*employed by for-profit companies: Jacob Beal is employed by Raytheon BBN Technologies; Markus*

*Gershater and Vishal Sanchania are employed by Synthace, and their work on this paper was thus*

*indirectly supported by their salaries. This does not alter the authors’ adherence to PLOS ONE policies*

*on sharing data and materials."*

*We note that one or more of the authors are employed by a commercial company: Raytheon BBN*

*Technologies and Synthace.*

*a) Please provide an amended Funding Statement declaring this commercial affiliation, as well as a*

*statement regarding the Role of Funders in your study. If the funding organization did not play a role in*

*the study design, data collection and analysis, decision to publish, or preparation of the manuscript and*

*only provided financial support in the form of authors' salaries and/or research materials, please review*

*your statements relating to the author contributions, and ensure you have specifically and accurately*

*indicated the role(s) that these authors had in your study. You can update author roles in the Author*

*Contributions section of the online submission form.*

*Please also include the following statement within your amended Funding Statement.*

*“The funder provided support in the form of salaries for authors [insert relevant initials], but did not*

*have any additional role in the study design, data collection and analysis, decision to publish, or*

*preparation of the manuscript. The specific roles of these authors are articulated in the ‘author*

*contributions’ section.”*

*If your commercial affiliation did play a role in your study, please state and explain this role within your*

*updated Funding Statement.*

*b) Please also provide an updated Competing Interests Statement declaring this commercial affiliation*

*along with any other relevant declarations relating to employment, consultancy, patents, products in*

*development, or marketed products, etc.*

*Please include both an updated Funding Statement and Competing Interests Statement in your cover*

*letter. We will change the online submission form on your behalf.*

*Please know it is PLOS ONE policy for corresponding authors to declare, on behalf of all authors, all*

*potential competing interests for the purposes of transparency. PLOS defines a competing interest as*

*anything that interferes with, or could reasonably be perceived as interfering with, the full and objective*

*presentation, peer review, editorial decision-making, or publication of research or non-research articles*

*submitted to one of the journals. Competing interests can be financial or non-financial, professional, or*

*personal. Competing interests can arise in relationship to an organization or another person. Please*

*follow this link to our website for more details on competing interests: http://journals.plos.org/*

*plosone/s/competing-interests*

To the best of our understanding of your request, the prior competing interest statement already satisfies

this request, because 1) it declares the employment affiliations and 2) there are no other financial interests

involved besides employment. If there is a further change required, please let us know.

Per the above request, please use the following expanded funding statement:

"Partial support for this work was provided by NSF Expeditions in Computing Program 469 Award

#1522074 as part of the Living Computing Project. Funder URL: https://www.nsf.gov/

The funders had no role in study design, data collection and analysis, decision to publish, or preparation

of the manuscript.

The following authors are employed by for-profit companies: Jacob Beal is employed by Raytheon BBN

Technologies; Markus Gershater and Vishal Sanchania are employed by Synthace. These companies

provided support in the form of salaries for these authors, but did not have any additional role in the study

design, data collection and analysis, decision to publish, or preparation of the manuscript. The specific

roles of these authors are articulated in the ‘author contributions’ section.”

*3. Please review your reference list to ensure that it is complete and correct. If you have cited papers that*

*have been retracted, please include the rationale for doing so in the manuscript text, or remove these*

*references and replace them with relevant current references. Any changes to the reference list should be*

*mentioned in the rebuttal letter that accompanies your revised manuscript. If you need to cite a retracted*

*article, indicate the article’s retracted status in the References list and also include a citation and full*

*reference for the retraction notice.*

To the best of our knowledge, our reference list is complete and correct and cites no retracted papers.

*4. Please consider amending the title to more accurately reflect the nature of the work. We note that*

*'meta-analysis' typically refers to an analysis following a systematic review and utilising a specific*

*framework.*

We have changed the term from "meta-analysis" to "comparative analysis", which we believe fits well.

*5. Please note that authors are responsible for ensuring that anyone named in the Acknowledgments*

*agrees to be named (https://journals.plos.org/plosone/s/submission-guidelines#loc-acknowledgments).*

All names listed in the acknowledgements section have been contributed from the participating

organizations via our consortium process.

**Reviewer #1:**

*In this manuscript, Beal et al conduct a metanalysis of fluorescence from bacterial strains. The data were*

*generated by participants in the 2016, 2017, and 2018 IGEM competitions. The analysis conducted by the*

*authors reveals high reproducibility in fluorescence across laboratories and years but also uncovers a*

*large anomaly in fluorescence measurements from 2016. The authors conduct further analysis and*

*suggest that the anomaly stems from errors in preparation of the fluorescent standard. The authors*

*conduct an experiment that supports this hypothesis. The authors conclude the article by providing*

*recommendations for investigators working in synthetic biology. Overall, this is a well-written article,*

*and the conclusions are generally supported by the data. By dealing with the often-overlooked issue of*

*experimental reproducibility, the study represents an important contribution to the field of synthetic*

*biology and should be accepted with only minor revisions.*

Thank you!

*We request that the authors consider addressing the following points:*

*Line 12: metrological traceability – unclear term – what does this mean to the reader not familiar with*

*the field of instrument calibration? It would be useful to define this term here.*

We have included the NIST definition: "establishment of an unbroken chain of calibrations to specified

reference standards"

*Line 98 – do you have a citation or reference for the statement that DH5-alpha and TOP10 are fairly*

*similar? How do you define “fairly similar”?*

We have added a pointer to public strain records at the Coli Genetic Stock Center.

*Line 105 – do the authors have a reference for the statement that FITC and fluorescein are near-identical*

*compounds with near-identical spectra.*

We have added a link to http://fluorophores.org/, one of multiple public databases containing this

information.

*Line 185 – “analysis of the 2018 study calibrant data finds this value to be 1.33e8” ... how did the*

*authors come up with this number?*

We computed this ratio by taking the ratio of the valid particles/Abs600 and OD/Abs600 conversion

factors that were computed for each team in the 2018 study, which produces units of particles/OD. We

have added this explanation at this point in the manuscript.

*Line 246-248 – can the authors speculate if there are particular factors that may contribute to the*

*remaining variation not explained by issues in fluorescence calibration?*

We have added the following speculation:

"We speculate that the remaining difference may be related to the low OD of the LUDOX HS-30 used in

2016, which would amplify the effect of any inaccuracy in the measurement of its reference value."

**Reviewer #2:**

*The manuscript by Beal and colleagues describes a meta-analysis of calibrated flow cytometry and plate*

*reader data from the iGEM inter-lab study across 2016, 2017 and 2018. The main results are that*

*calibration of both data types provides reproducibility across laboratories and machines. This message is*

*very important for the synthetic biology field if it is really going to mature into a true engineering*

*discipline. Another point from the paper is that errors in the calibration protocol can be disastrous for*

*metrology and it is interesting to see the possible impact.*

*Overall, I think the authors have done a thorough job in collating the data across years and the message*

*of the paper should (hopefully) have a big impact in the field.*

Thank you!

*I have one minor comment: When talking about the different protocols across years, the authors say the*

*results across years are expected to be equivalent, and thus directly comparable. While this may be true*

*for the expected values, it is not obvious to me that the noise distributions should be the same and*

*changing concentrations etc will affect the variability. Can the authors add a comment on this?*

If there are any differences in noise distributions or protocol variability, these should be observable as

differences in the distributions of calibrant and cell measurements. We have added another bullet to this

effect in the discussion of protocol differences.

In fact, of course, the data presented in Figure 2 and Figure 3 show that the calibrants have quite

consistent levels of variability from year to year, and Figure 4 shows that the team-to-team noise

distribution is consistent from year to year as well.
